# Glycated haemoglobin threshold for dysglycaemia screening, and application to metabolic syndrome diagnosis in HIV-infected Africans

**DOI:** 10.1371/journal.pone.0211483

**Published:** 2019-01-31

**Authors:** Kim A. Nguyen, Nasheeta Peer, Anniza de Villiers, Barbara Mukasa, Tandi E. Matsha, Edward J. Mills, Andre P. Kengne

**Affiliations:** 1 Non-Communicable Diseases Research Unit, South African Medical Research Council, Cape Town, South Africa; 2 Department of Medicine, Faculty of Health Sciences, University of Cape Town, Cape Town, South Africa; 3 Centre for Evidence-based Health Care, Division of Epidemiology and Biostatics, Faculty of Medicine and Health Sciences, Stellenbosch University, Cape Town, South Africa; 4 United Nations Population Fund (UNFPA), Mildmay, Uganda; 5 Department of Biomedical Sciences, Faculty of Health and Wellness Science, Cape Peninsula University of Technology, Cape Town, South Africa; 6 Global Evaluation Science, Vancouver, Canada; Florida International University Herbert Wertheim College of Medicine, UNITED STATES

## Abstract

**Background:**

Glycated haemoglobin (HbA1c) test has been increasingly promoted as an alternative to fasting plasma glucose (FPG) or oral glucose tolerance test (OGTT) to diagnose dysglycaemia but its performance in HIV-infected Africans has yet to be established. This study aimed to assess the diagnostic accuracy of HbA1c for dysglycaemia including FPG-defined and OGTT-defined dysglycaemia, and OGTT-defined diabetes in HIV-infected Africans, and the effect of HbA1c-predicted dysglycaemia on Joint Interim Statement (JIS)-based prevalent metabolic syndrome (MS).

**Methods:**

A cross-sectional study included HIV-positive patients recruited across public healthcare facilities in the Western Cape. The recommended HbA1c cut-points were tested alongside the optimal cut-points obtained from receiver operating characteristic curve analyses, while the agreement between the MS criteria were assessed using kappa statistic.

**Results:**

748 participants (157 men), median age 38 years, 93% on anti-retroviral drugs were included. The optimal HbA1c cut-points of 5.75% (39.3 mmol/mol) showed 54% sensitivity, 84% specificity for FPG-defined dysglycaemia, and 52% sensitivity, 85% specificity for OGTT-defined dysglycaemia. The HbA1c value of 5.85% (40.4 mmol/mol) (63% sensitivity, 99% specificity) was optimal for diabetes. The internationally advocated cut-point of 6.5% (48 mmol/mol) had 37% sensitivity and 99% specificity for diabetes, while HbA1c ≥5.7% (≥39 mmol/mol) yielded similar performance with the study-specific cut-point for any dysglycaemia. MS prevalence by the JIS criteria (28.2%) increased to 29.7% when using HbA1c ≥5.75% (≥39.3 mmol/mol) and to 32.9% with HbA1c ≥5.7% (≥39 mmol/mol); agreement between the original and modified criteria was generally good.

**Conclusions:**

This study agrees with the internationally recommended HbA1c cut-point for detecting dysglycaemia, but not for diabetes in HIV-infected Africans. In line with previous studies in general African populations, our findings suggest that similar factors interfere with HbA1c values regardless of HIV infection status. Replacing FPG-based with HbA1c-predicted dysglycaemia in the JIS criteria to diagnose MS is feasible in HIV-infected Africans.

## Introduction

Measuring fasting plasma glucose (FPG) levels or performing an oral glucose tolerance test (OGTT), are the currently recommended tools for diagnosing diabetes and other categories of dysglycaemia [1,2]. However, these tests are inconvenient requiring an overnight fast and the OGTT is cumbersome as it necessitates a 2-hour waiting period. Given these inconveniences and the day-to-day variability in glucose, there is a need for a reliable, high performance, convenient and low-cost alternative. Glycated haemoglobin (HbA1c), which reflects the average plasma glucose concentration over the previous 8–12 weeks, has been used in diabetes care to monitor glucose control [3]. Notably, it has also been suggested for use as an alternate diagnostic tool [1]. Previously, the test was expensive and there were concerns about accuracy of measurements. However, since 2009, with advances in technology, assay standardisation and costs have improved. HbA1c affords the convenience of not requiring an overnight fast nor a waiting period. It can be performed at any time of the day and overcomes the day-to-day variability in glucose levels [3]. Consequently, HbA1c is now increasingly considered for use as a diagnostic tool for diabetes and high-risk of diabetes [1,2].

The use of a convenient test that does not require pre-planning nor a waiting period would be particularly advantageous in Sub-Saharan Africa where there are numerous barriers to healthcare service access. These include travelling long distances and high out-of-pocket expenses which prevent revisits, particularly at short intervals, and consequently, a large proportion of individuals with diabetes remain undiagnosed in the region [4]. However, the ability of HbA1c to diagnose dysglycaemia in African populations has been variable [5]. Haemoglobinopathies, anaemia and haemolysis influencing the accuracy of HbA1c results are frequent in Africa. Furthermore, the burden of HIV infection is high in Africa, and anaemia and haemolysis are more common in HIV-infected individuals compared with the general population [6,7]. However, the ability of HbA1c to accurately diagnose diabetes and other dysglycaemias in HIV-infected individuals has not yet been established.

Therefore, in the current study, we assessed the accuracy of HbA1c for diagnosing any dysglycaemia (impaired fasting glycaemia and/or impaired glucose tolerance) and screen-detected diabetes in a population of South Africans living with HIV infection. Additionally, we assessed the prevalence of metabolic syndrome (MS), defined by the Joint Interim Statement (JIS) criteria, when HbA1c was used to diagnose hyperglycaemia instead of fasting glucose.

## Materials and methods

### Design and population

The current study is based on cross-sectional data collected between March 2014 and February 2015; the methodological approach has been described in detail elsewhere [8]. In brief the participants were recruited from public healthcare facilities in Cape Town (10) and the surrounding rural municipalities (seven) where the majority of residents were black and coloured [9], using simple random sampling procedures without any stratification. Consenting HIV-positive men and women aged 18 years or older, and were not pregnant, breastfeeding, bedridden, undergoing treatment for cancer, nor on corticosteroid treatment were included.

The study was approved by the South African Medical Research Council Ethics Committee, according to Official Letter no. EC021-11/2013, and by the Health Research Office of the Western Cape Department of Health, document no. RP 005/2014. All participants signed informed consent forms prior to the study procedure. Data sharing was not included in the consent.

### Data collection

The data were collected by a well-trained research team including clinicians, nurses and fieldworkers, and captured on personal digital assistants (PDAs) onto a web-based respondent driven sampling research management system [10]. This system used electronic case report forms with built-in checks for quality control to ensure the quality and integrity of the data collected in real-time. At the same time, the system allowed the participant’s data to be linked and tracked throughout the research site via a unique barcode using BRYANT Research systems software [10]. Data on socio-demographic and medical history were obtained from a structured interviewer-administered questionnaire adapted from the WHO STEPwise approach to Surveillance (STEPS) tool (S1 File). HIV-related information such as duration of diagnosed HIV infection, CD4 counts and antiretroviral therapy (ART) regimens were from the participants’ records.

#### Measurements

Anthropometry was done using standardised techniques. Heights and weights were measured with the participants in light clothing and without shoes. Blood pressure (BP) were taken on the right arm, using a digital BP monitor (Omron, M6 Comfort, Netherland) after seating the participant in a resting position for at least five minutes. Three BP measurements were taken three minutes apart, and the average of 2^nd^ and 3^rd^ readings was used in the analysis.

All participants who did not have a history of diabetes underwent a standard 2-hour 75 grams OGTT after an overnight fast. Plasma glucose levels were determined at fasting (FPG) and at 2-hour post-OGTT (2h-PG). Blood samples were drawn and processed for laboratory analyses. The concentrations of glucose and lipid were measured with an autoanalyser, Beckman Coulter AU 500 spectrophotometer, while hexokinase and enzymatic colorimetric methods were used to analyse plasma glucose and serum triglycerides and high density lipoprotein cholesterol (HDL-C) respectively. HbA1c was measured using high-performance liquid chromatography (VARIANT II TURBO, EDTA tubes) in accordance with the National Glycohaemoglobin Standardisation Programme (NGSP).

#### Definitions

The following dysglycaemia categories were defined: Raised FPG: FPG≥5.6 mmol/L, raised 2h-PG: 2h-PG ≥7.8 mmol/L, and diabetes or screen-detected diabetes as FPG≥7.0 mmol/L and/or 2h-PG≥11.1 mmol/L without previously diagnosed diabetes [11]. Participants with previously diagnosed diabetes were excluded from the data analyses.

MS components and their cutoffs were defined based on JIS criteria: increased waist circumference (WC): men≥94 cm, women ≥80 cm; high triglycerides: ≥1.7 mmol/L; low HDL-C: men<1.03 mmol/L, women<1.3 mmol/L; raised BP: ≥130/85 mmHg or on hypertensive medication; hyperglycemia: FPG≥5.6 mmol/L or on glucose control agents [12].

### Statistical analysis

The R statistical software version 3.3.1 (2016-06-21), (The R Foundation for Statistical Computing Platform, Vienna, Austria) was used for data analyses. Continuous data are presented as means (± standard deviation, SD) or medians (25^th^-75^th^ percentiles), while categorical data as frequencies and percentages. Mann-Whitney U test and chi square test were used to compare men vs. women data. Kappa statistic was computed to assess the concordance between the diagnostic criteria of MS: the JIS and those modified using HbA1c instead of FPG. The kappa values are interpreted as poor (kappa≤0.2), fair (kappa≤0.4), moderate (kappa≤0.6), substantial (kappa≤0.8), and very good (kappa>0.8).

The receiver operating characteristic curves (ROC) analyses were performed using the “pROC” package. The optimal cut-point level of HbA1c was determined using two methods: 1) the closest top-left point and 2) the Youden’s index (J-point). 1) In the ROC analysis, pairs of the true positive rate (sensitivity) and the false positive rate (1-specificity) and for every individual cut-point are plotted. The shape of the ROC curve indicates the discriminative power level of the test, and perfect discrimination is where the ROC curve passes through the upper-left corner (100% sensitivity, 100% specificity). Therefore, when the ROC curve is closer to the upper-left corner and the area under the curve (AUC) is larger, the overall accuracy of the test is higher, and the cut-point nearest to the upper-left corner is defined as the optimal one. The Youden’s index is computed as sensitivity + specificity– 1 and ranges from 0 to 1. Maximising this index (J-point) allows finding an optimal cut-point independently from the outcome prevalence. The 95% confidence interval of the derived optimal cut-point was computed using bootstrap sampling based on 2000 replicates.

The diagnostic accuracy of the derived cut-off level was assessed alongside American Diabetes Association (ADA) / International Diabetes Federation (IDF) recommended thresholds by computing a number of diagnostic performance measures including the *sensitivity* which is the probability that a person has a positive test result given that of having a positive outcome; the *specificity*, the probability that a person has a negative test result given that of having a negative outcome; the *positive predictive value* (PPV), the probability that a person has a positive outcome given that of having a positive test result; the *negative predictive value* (NPV), the probability that a person has a negative outcome given that of having a negative test result, and the *Youden’s index*. All these calculations were done with the “epiR” package of R.

## Results

### General characteristics of the participants

**Fig 1:**
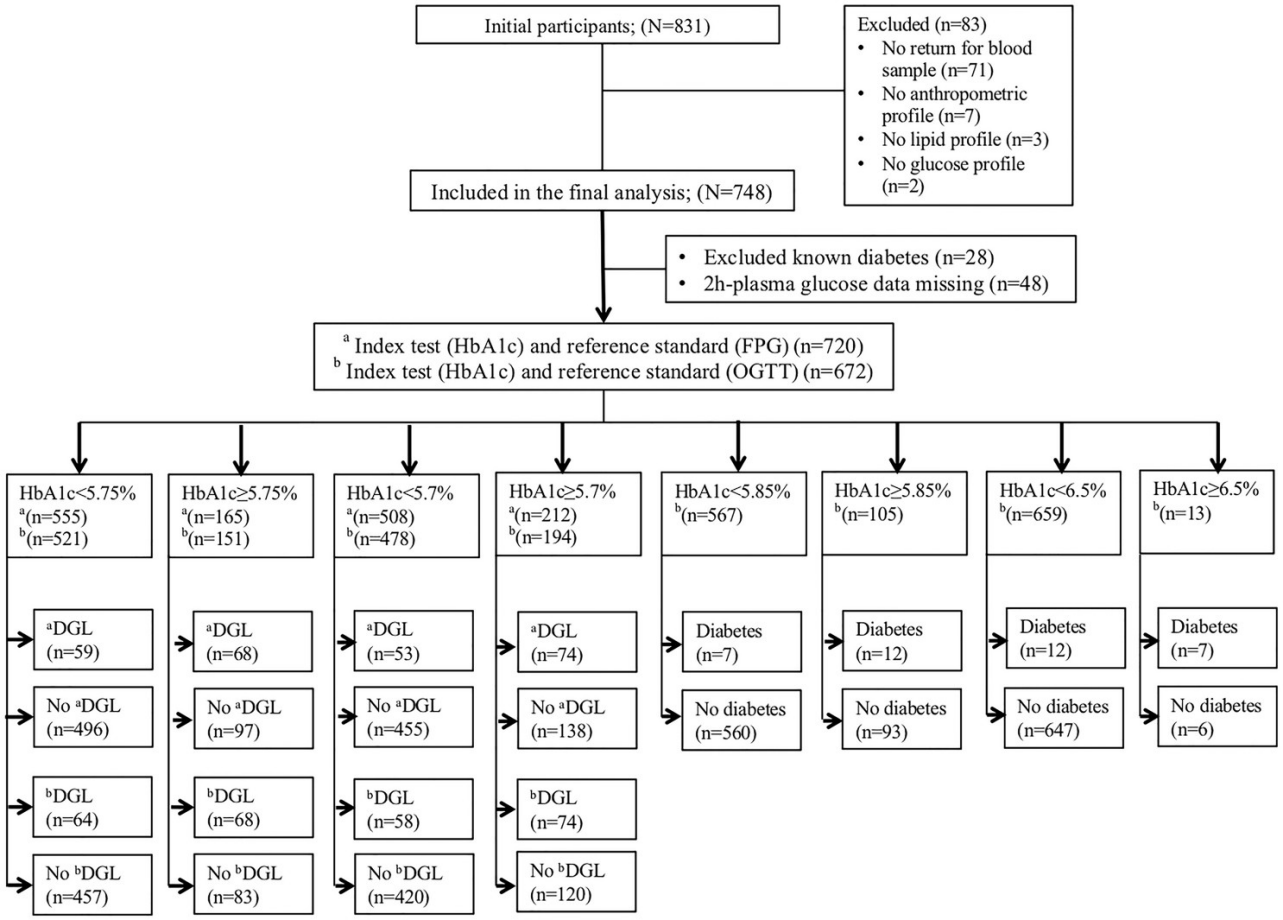
STARD diagram describes the flow of the participants throughout the study analyses. ^a^DGL, dysglycaemia is based on fasting plasma glucose (FPG) ≥5.6 mmol/L; ^b^DGL is based on FPG ≥5.6 mmol/L and/or 2h-plasma glucose≥7.8 mmol/L; Diabetes is defined as World Health Organization criteria (FPG ≥7.0 mmol/L and/or 2h-plasma glucose ≥11.1 mmol/L).

Fig 1, which is the Standard of Reporting for Diagnostic Accuracy Studies (STARD) diagram, demonstrates the flow of participants in the present study. The starting sample comprised 831 participants of which 83 had missing data on at least one component of the JIS-defined MS, and were excluded. Therefore, the main analytic sample comprised 748 participants including 157 men and 591 women. Of these, 48 with missing 2h-PG data were excluded from the OGTT-related analyses.

The clinical and biochemical characteristics of the participants are summarised in Table 1. Their median age was 38 years (25^th^-75^th^ percentiles: 35–42), and 93% were ART users. The median CD4 count was 392 cells/mm^3^ (25^th^-75^th^ percentiles: 240–604) and the median duration of diagnosed HIV infection was 5 years (25^th^-75^th^ percentiles: 2–9). Women had higher CD4 counts and longer duration of diagnosed HIV infection than men (both p≤0.001). The median HbA1c was 5.5% (25^th^-75^th^ percentiles: 5.2–5.8) [37 mmol/mol (25^th^-75^th^ percentiles: 33–40)] with no difference between men and women (p = 0.344). Furthermore, women had greater WC, BMI, systolic BP, higher levels of HDL-C, high-sensitivity C-reactive protein (hs-CRP), but lower values of triglycerides, gamma-glutamyl transferase (gamma-GT) as well as serum creatinine compared to men (all p≤0.023). The overall prevalence of dysglycaemia was 17.6% based on FPG alone, and 19.6% based on OGTT. Nineteen participants (2.8%) were identified with screen-detected diabetes; while 28 (3.7%) participants had known diabetes. The prevalence of dysglycaemia, screen-detected diabetes and previously diagnosed diabetes did not vary significantly among men and women (all p≥0.305).

**Table 1:** Cardio-metabolic risk and HIV-related characteristics in men and women.

Characteristics	Overall (N = 748)	Men (n = 157)	Women (n = 591)	P-value
Median (25^th^–75^th^ percentiles)				
Age (years)	38 (32–44)	41 (35–47)	37 (31–43)	<0.001
Known diabetes, n (%)	28 (3.7)	8 (5.1)	20 (3.4)	0.315
Waist circumference (cm)	88 (78–98)	78.9 (74–88)	90 (80–101)	<0.001
Body mass index (kg/m^2^)	26.3 (22.1–32)	21.4 (19.8–22.4)	28.3 (23.8–28.9)	<0.001
Systolic blood pressure (mmHg)	117 (107–130)	123.5 (114.5–140)	115 (105.8–127)	<0.001
Diastolic blood pressure (mmHg)	82 (75–91)	83 (76–94)	81.5 (74.8–89.8)	0.129
HbA1c (%) (mmol/mol)	5.5 (5.2–5.8) 37 (33–40)	5.5 (5.2–5.8) 37 (33–40)	5.4 (5.2–5.7) 36 (33–39)	0.344
Fasting plasma glucose (mmol/L)	5 (4.6–5.4)	5.1 (4.8–5.5)	4.9 (4.6–5.4)	0.010
2h-plasma glucose (mmol/L)	5.3 (4.6–6.2)	5.15 (4.4–6.3)	5.4 (4.6–6.2)	0.262
FPG-based dysglycemia^1^, n (%)	127/720 (17.6)	28/149 (18.8)	99/571 (17.3)	0.678
OGTT based dysglycemia^2^, n (%)	132/672 (19.6)	32/141 (22.7)	100/531 (18.8)	0.305
Diabetes^3^, n (%)	19 (2.8)	2 (1.4)	17 (3.2)	0.392
Triglycerides (mmol/L)	1 (0.7–1.3)	1.12 (0.75–1.27)	0.97 (0.74–1.28)	0.023
HDL-C (mmol/L)	1.3 (1–1.5)	1.2 (1.0–1.5)	1.29 (1.08–1.52)	0.010
hs-CRP (mg/L)	5.6 (2.4–14.5)	4.9 (2.1–16.2)	5.6 (2.4–14.2)	0.728
gamma-GT (IU/L)	39 (26–66)	53 (30–96)	38 (25–58)	<0.001
Creatinine (*μ*mol/L)	58 (51–67)	70 (61–79)	56 (49–62)	<0.001
HIV duration (years)	5 (2–9)	4 (2–7)	5 (2.5–9)	<0.001
CD4 count (cells/mm^3^)	392(240–604)	272 (193–448)	410 (253–627)	0.001
ART-usage, n (%)				0.296
ART-naive	46/699 (6.6)	7/149 (4.7)	39/550 (7.1)	
ART-treated	653/699 (93.4)	142/149 (95.3)	511/550 (92.9)	

ART, antiretroviral therapy; BMI, body mass index; FPG, fasting plasma glucose; HDL-C, high-density lipoprotein cholesterol; HIV, human immunodeficiency virus; hs-CRP, high sensitivity C-reactive protein; gamma-GT, gamma-glutamyl transferase;

^1^FPG ≥5.6 mmol/L;

^2^FPG ≥5.6 mmol/L and/or 2h-plasma glucose ≥7.0 mmol/L;

^3^FPG ≥7.0 mmol/L and/or 2h-plasma glucose ≥11.1 mmol/L without previously diagnosed diabetes.

### Optimal cut-point of HbA1c for dysglycaemia and diabetes

**Fig 2:**
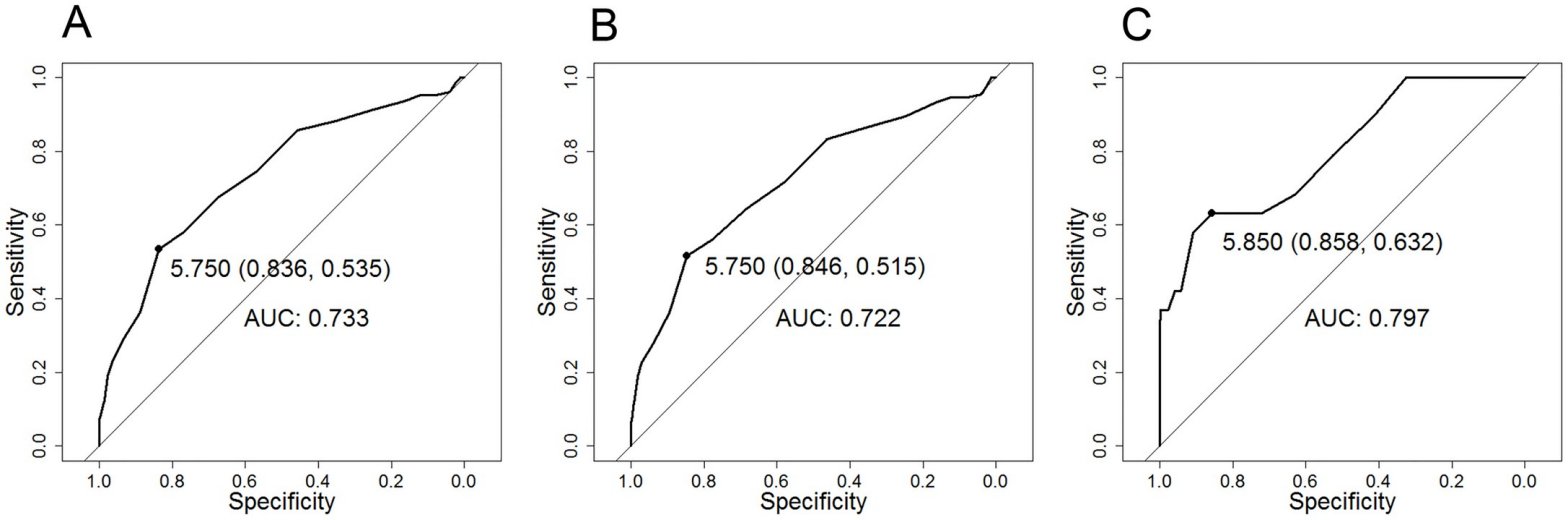
ROC curve characteristics of HbA1c that corresponded with fasting plasma glucose (FPG) ≥5.6 mmol/L (A), FPG ≥5.6mmol/L or 2-hour oral glucose tolerance test (OGTT) ≥7.8mmolL (B), and FPG ≥7.0mmol/L and 2-hour OGTT ≥11.1mmol/L (C) in HIV-infected participants without known diabetes. The ROC curves show the same optimal cut-point of HbA1c of 5.75% (39.3 mmol/mol) for diagnosing dysglycaemia based on FPG (A) (AUC: 0.733, sensitivity: 54%, specificity: 84%) or based on OGTT (B) (AUC: 0.722, sensitivity: 52%, specificity: 85%), and the optimal HbA1c of 5.85% (40.4 mmol/mol) for screen-detected diabetes (C) (AUC: 0.797, sensitivity: 63%, specificity: 86%).

The AUCs for HbA1c to identify participants with dysglycaemia was 0.733 (95% confidence interval [CI]: 0.682–0.784) for FPG-diagnosed dysglycaemia and 0.722 (95%CI: 0.670–0.774) for OGTT-diagnosed dysglycaemia (Fig 2). The optimal HbA1c cut-point for either FPG or OGTT-diagnosed dysglycaemia was 5.75% (95%CI: 5.35–5.75) [39.3 mmol/mol (35–39.3)]. Table 2 shows the performance of different HbA1c cut-points for detecting dysglycaemia and screen-detected diabetes among participants. For FPG-defined dysglycaemia, the derived cut-point was 5.75% (39.3 mmol/mol) with the following performance measures: sensitivity 54% (95%CI: 44–62), specificity 84% (80–87), Youden’s index 0.37 (0.25–0.49), PPV 41% (34–49), and NPV 89% (87–92). The cut-point 5.7% (39 mmol/mol), which is recommended by the ADA and the IDF, showed a sensitivity of 58% (49–67), specificity 77% (73–80), Youden’s index 0.35 (0.22–0.47), PPV 35% (29–42), and NPV 0.90 (0.87–0.92). For OGTT-defined dysglycaemia, the derived HbA1c cut-point of 5.75% (39.3 mmol/mol) yielded a sensitivity of 52% (43–60), specificity 85% (81–88), Youden’s index 0.37 (0.23–0.45), PPV 48% (40–56), and NPV 85% (82–88) while the cut-point of 5.7% (39 mmol/mol) gave a sensitivity of 56% (47–65), specificity 78% (74–81), Youden’s index 0.34 (0.21–0.46), PPV 38% (31–45), and NPV 88% (85–91).

**Table 2:** Performances of HbA1c corresponding with dysglycaemia, and screen-detected diabetes among the participants without history of diabetes from ROC curve analysis.

Outcome measured	HbA1c cut-point (95%CI)		AUC (95%CI)	Sensitivity (95%CI)	Specificity (95%CI)	Youden-Index (95%CI)	PPV (95%CI)	NPV (95%CI)
mmol/L	%	mmol/mol						
FPG≥5.6	5.7	39		0.58 (0.49–0.67)	0.77 (0.73–0.80)	0.35 (0.22–0.47)	0.35 (0.29–0.42)	0.90 (0.87–0.92)
	5.75 (5.35–5.75)	39.3 (35–39.3)	0.733 (0.682–0.784)	0.54 (0.44–0.62)	0.84 (0.80–0.87)	0.37 (0.25–0.49)	0.41 (0.34–0.49)	0.89 (0.87–0.92)
FPG≥5.6 and/or 2h-glucose≥7.8	5.7	39		0.56 (0.47–0.65)	0.78 (0.74–0.81)	0.34 (0.21–0.46)	0.38 (0.31–0.45)	0.88 (0.85–0.91)
	5.75 (5.35–5.75)	39.3 (35–39.3)	0.722 (0.670–0.774)	0.52 (0.42–0.58)	0.85 (0.81–0.88)	0.37 (0.23–0.45)	0.48 (0.40–0.56)	0.85 (0.82–0.88)
FPG≥7.0 and/or 2h-glucose≥11.1	5.85 (5.25–6.65)	40.4 (33.9–49.2)	0.797 (0.686–0.907)	0.63 (0.38–0.84)	0.86 (0.83–0.88)	0.49 (0.22–0.73)	0.11 (0.06–0.19)	0.99 (0.97–1.00)
	6.5	48		0.37 (0.16–0.62)	0.99 (0.98–1.00)	0.36 (0.22–0.80)	0.55 (0.30–0.80)	0.98 (0.97–0.99)

FPG, fasting plasma glucose; AUC, area under the curve; Sensitivity = TP/(TP+FN); Specificity = TN/(TN+FP); Youden’s index = (sensitivity + specificity)– 1; PPV, positive predictive value = TP/(TP+FP); NPV, negative predictive value = TN/(TN+FN); where TP, true positive; FP, false positive; TN, true negative; FN, false negative.

The AUC of HbA1c to diagnose participants with screen-detected diabetes was 0.797 (0.686–0.907) (Fig 2). The optimal cut-point was 5.85% (5.25–6.65) [40.4 mmol/mol (33.9–49.2)], and the resultant performance measures were: sensitivity 63% (38–84), specificity 86% (83–88), Youden’s index 0.49 (0.22–0.73), PPV 11% (6–19), and NPV 99% (97–100), (Table 2). The HbA1c cut-point of 6.5% (48 mmol/mol), recommended by the ADA and IDF had a sensitivity of 37% (16–62), specificity 99% (98–100), Youden’s index 0.36 (0.22–0.80), PPV 55% (30–80), and NPV 98% (97–99), Table 2.

### Prevalence of MS using FPG or HbA1c as the hyperglycaemia criterion

**Fig 3:**
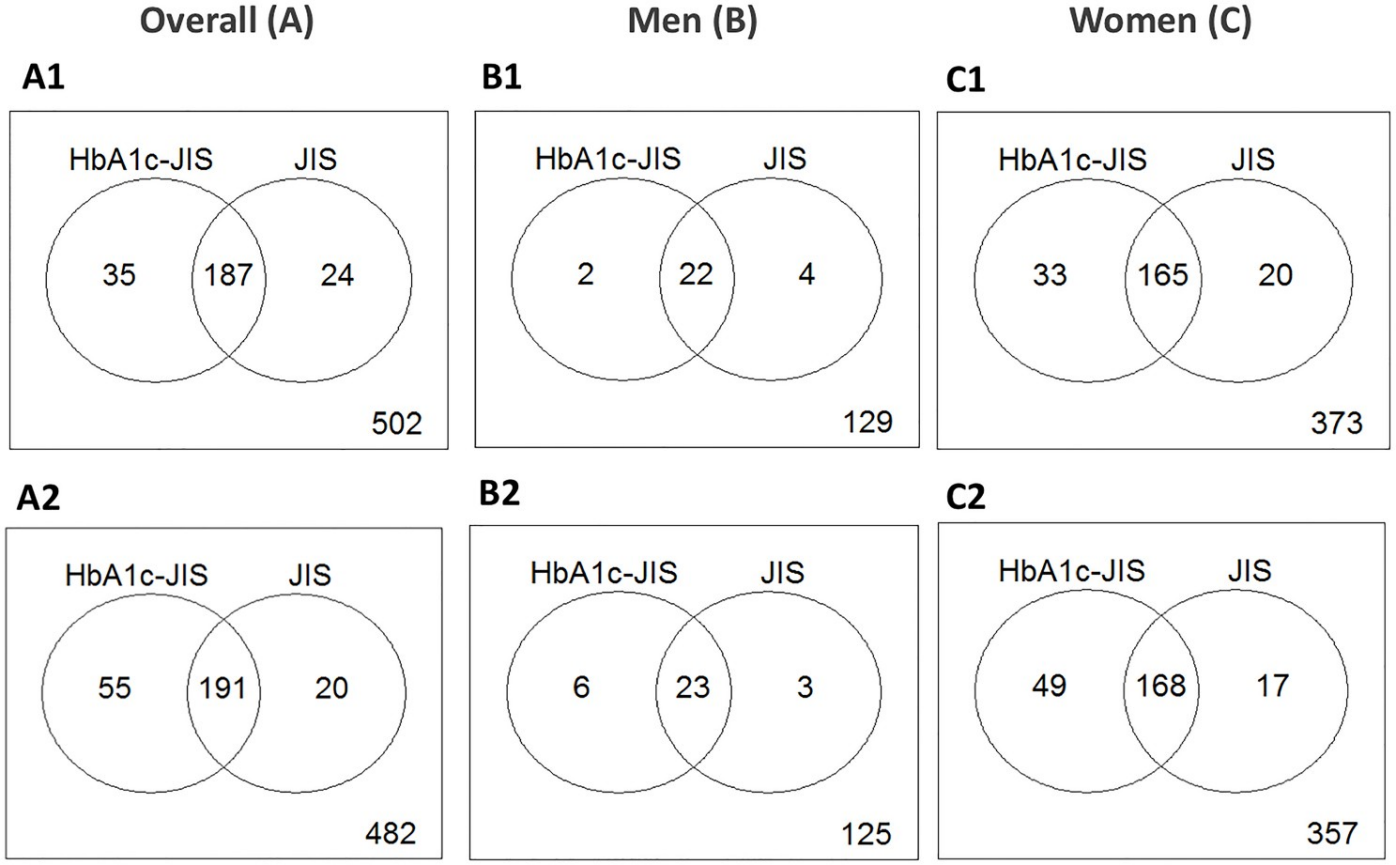
Metabolic syndrome by the Joint Interim Statement criteria: Comparing prevalence using dysglycaemia criteria of fasting plasma glucose with HbA1c in participants without knowing diabetes. The first row shows prevalence of MS based on JIS-HbA1c ≥5.75% (≥39.3 mmol/mol) and JIS criteria, overall (A1), men (B1), women (C1); the second row shows the prevalence of MS based on JIS-HbA1c ≥5.7% (≥39 mmol/mol) and JIS criteria, overall (A2), men (B2), women (C2).

Fig 3 depicts the prevalence of MS according to the original and modified JIS using HbA1c cut-points. Based on the original JIS criteria which uses FPG≥5.6 mmol/L, the prevalence of MS was 28.2% (211/748) overall, 16.6% (26/157) in men, and 31.3% (185/591) in women (p<0.001). Replacing FPG with HbA1c≥5.75% (≥39.3 mmol/mol) yielded the prevalence of 29.7% (222/748) overall, 15.3% (24/157) in men, and 33.5% (198/591) in women (p<0.001). Out of 246 participants who were diagnosed with the MS based on either FPG or HbA1c ≥5.75% (≥39.3 mmol/mol)], 187 (76%) were identified by both criteria, 35 participants (14.2%) met the HbA1c criteria only while 24 (9.7%) participants met the FPG criteria only, [kappa = 0.81 (95%CI: 0.76–0.86)].

If HbA1c ≥5.7% (≥39 mmol/mol) was used, the MS prevalence would be 32.9% (246/748) overall, 18.5% (29/157) in men, and 36.7% (217/591) in women (p<0.001). Among 266 participants with MS according to either FPG or HbA1c [cut-point 5.7% (39 mmol/mol)], 72% were diagnosed by both criteria, 20.7% by only HbA1c and 7.5% by FPG, [kappa = 0.76 (0.71–0.81)]. Fig 3.

## Discussion

The present study is among the first to examine the performance of HbA1c as a diagnostic test for dysglycaemia or diabetes in a sub-Saharan African population living with HIV infection. Our key findings are the following: 1) HbA1c had an acceptable-to-good discriminatory ability to detect prevalent dysglycaemia (impaired fasting glycaemia, and/or impaired glucose tolerance) and screen-detected diabetes; 2) The study-specific optimal HbA1c cut-point to detect the presence of dysglycaemia was not appreciably different from the advocated cut-point by the ADA and IDF, while the optimal cut-point to detect screen-detected diabetes was lower than that recommended by the two organisations, but in line with previous studies in the general population in Africa [5, 13]; 3) Replacing FPG-based with HbA1c-predicted dysglycaemia in the JIS MS criteria led to marginally higher prevalence estimates, with generally good agreement between the original JIS and the modified criteria.

Although HbA1c has been recommended by the ADA and IDF as an alternative test for diagnosing diabetes and individuals at high risk for diabetes, its applicability and suitable thresholds in various populations remain unresolved [14]. In the present study, the optimal cut-point of HbA1c for detecting diabetes was lower than the one recommended by the ADA/IDF but within the range of the cut-point found in a mixed-ancestry South African population [5]. The data in that study were from 819 participants with median age of 52 and residing in the local community [5]. We found no similar data from HIV-infected Africans for direct comparison. Nonetheless, our findings agreed with a study from the United State which showed that the HbA1c threshold of 6.5% (48 mmol/mol) was insensitive but highly specific while the HbA1c level of 5.8% (40 mmol/mol) was ideal for diagnosing diabetes in HIV-infected patients [15]. Furthermore, a pooled analysis of data from 96 population-based health examination surveys yielded HbA1c 6·5% or more had a pooled sensitivity of 30·5% compared with FPG or-2hOGTT-based diabetes, with the heterogeneity that could not explained by the preselected study-level characteristics [14].

For the detection of dysglycaemia, the derived HbA1c cut-point of 5.75% (39.3 mmol/mol) is similar to that found in mixed-ancestry South Africans [13] and not appreciably different from the 5.7% (39 mmol/mol) recommended by the ADA/IDF. It should be understood that the present and previous studies in South Africa tend to agree more on HbA1c-based diagnosis for dysglycaemia than diabetes. It has been reported that HbA1c may not accurately reflect glycaemia in individuals with abnormal haemoglobin [16]. Iron and vitamin B12 deficiency with and without anaemia have been reported to reduce erythropoiesis, and thus reduce erythrocyte turnover which lead to increase in HbA1c values independently of glucose levels [16]. Some studies have suggested that low-grade haemolysis might contribute to lower HbA1c value at a given glucose level in HIV-infected patients than HIV-uninfected individuals [6,17]. Despite the above, the derived cut-points in our study appear consistent with those obtained in the local general population [5,13]. This suggests that the factors that influence HbA1c values in African populations are unlikely to differ by HIV status. In part, this similarity could be explained by relatively short duration of HIV infection (median diagnosed HIV was 5 years), and successful HIV treatment (majority of the participants was on first-line ART regimen) among the present study participants. Nonetheless, small sample size preclude reliable analyses stratified by CD4 count.

Replacing FPG with HbA1c showed the change in MS prevalence to be marginal, with HbA1c diagnosing slightly more people than FPG while missing only a tiny proportion diagnosed by FPG in the present study. This suggests that HbA1c could be used as the hyperglycaemia criterion for MS in HIV-infected individuals. This finding is essential in African populations, especially people infected with HIV. Although MS definitions have used FPG as the diagnostic criterion for hyperglycaemia, African studies have found FPG alone to be an inadequate screening test for dysglycaemia or diabetes in general populations since it misses a significant proportion of individuals who tend to only have 2-hour abnormalities [18]. Replacing FPG with HbA1c to identify hyperglycaemia or diabetes in the diagnosis of MS in African populations has relevance as HbA1c could possibly identify individuals who may also have dysglycaemia on the 2-hour OGTT while overcoming the challenges of performing the OGTT in these specific populations. This is particularly relevant for Africans living with HIV infection who regularly require routine screening for cardiovascular health. Indeed, the requirement for an overnight fasting and the long waiting periods for the completion of the OGTT would be problematic for both HIV-care providers and the patients.

### Strengths and limitations

Our study had some limitations with the wide confidence interval of the optimal HbA1c cut-point for diagnosing diabetes indicating a lack of statistical power due to the small sample size. The absence of an HIV-uninfected group and of external validation limit the recommendation of our results for application in routine setting. Another limitation was that data on erythrocyte abnormalities were not collected in the present study. Nonetheless, our study has numerous strengths. Apart from a multiple-clinic study, this is the first to examine the performance of HbA1c as a diagnostic test for glycaemic disorders and the MS in a sub-Sahara African population living with HIV infection. Another strength was the availability of not only FPG but also 2h-PG levels for the analyses of HbA1c cut-points corresponding to both FPG and 2h-OGTT.

## Conclusions

In this HIV-infected African population, the optimal HbA1c cut-point to detect the presence of dysglycaemia was not appreciably different from the advocated cut-point by the ADA and IDF, while the optimal cut-point to detect screen-detected diabetes was lower than that recommended by the two organisations. Importantly, these findings are in line with previous studies in the general population in Africa, suggesting that factors influencing HbA1c values are likely to be similar in African HIV-infected and uninfected populations. Our study findings further support that replacing the FPG criterion in the JIS MS definition with HbA1c will have only marginal effects on MS prevalence, while facilitating the screening of the condition. However, these findings need to be confirmed by other studies in HIV-infected African populations. Ideally, such studies should be nested with interventions to mitigate the risk, using evidence generated from the general population.

## Supporting information

S1 File Self-reported physical and medical history. (DOCX)
