# Supplementary material for: Glycated haemoglobin threshold for dysglycaemia screening, and application to metabolic syndrome diagnosis in HIV-infected Africans
Source: PLoS One. 2019 Jan 31;14(1):e0211483. doi: 10.1371/journal.pone.0211483 (PMC6355005; doi:10.1371/journal.pone.0211483)
Supplement: S1 File — (DOCX) [file pone.0211483.s001.docx]

**SELF-REPORTED PHYSICAL and MEDICAL HISTORY**

| **Q1** **Would you say your health is** | | | | | | | | | | | | | | | | | | | | | | | | | | | | | | | | |
| --- | --- | --- | --- | --- | --- | --- | --- | --- | --- | --- | --- | --- | --- | --- | --- | --- | --- | --- | --- | --- | --- | --- | --- | --- | --- | --- | --- | --- | --- | --- | --- | --- |
| Poor | | | | |  | | | | | | |  | | | | | | | | | | | | | | | | | | | |  |
| Fair | | | | |  | | | | | | |  | | | | | | | | | | | | | | | | | | | |  |
| Good | | | | |  | | | | | | |  | | | | | | | | | | | | | | | | | | | |  |
| Very Good | | | | |  | | | | | | |  | | | | | | | | | | | | | | | | | | | |  |
| Excellent | | | | |  | | | | | | |  | | | | | | | | | | | | | | | | | | | |  |
| **Q2** **Do you personally think that you are** DON'T KNOW (DK) | | | | | | | | | | | | | | | | | | | | | | | | | | | | | | *DK* | | |
| Underweight | | | | |  | | | | | | |  | | | | | | | | | | | | | | | | | | | |  |
| Normal Weight | | | | |  | | | | | | |  | | | | | | | | | | | | | | | | | | | |  |
| Overweight | | | | |  | | | | | | |  | | | | | | | | | | | | | | | | | | | |  |
| **HIV AND NCD HISTORY: The next few questions relate to HIV**  **Q3 How long ago were you diagnosed HIV positive?** *COMPLETE WHAT IS APPLICABLE, E.G. 2 YRs 6 MNTHs* | | | | | | | | | | | | | | | | | | | | | | | | | | | | | | *DK* | | |
| Years | | | | |  | | |  | | | | | |  | | | | | | | | | | | | | | | |  | |  |
| Months | | | | |  | | |  | | | | | |  | | | | | | | | | | | | | | | |  | |  |
| Weeks | | | | |  | | |  | | | | | |  | | | | | | | | | | | | | | | |  | |  |
| Days | | | | |  | | |  | | | | | |  | | | | | | | | | | | | | | | |  | |  |
| **Q4 Do you know what your latest CD4 count is?** | | | | | | | | | | | | | | | | | | | | | | | | | | | | | | | | |
| I know my latest CD4: | | | | | | | | | | | | | | | | | |  | |  | | | | | | | | | | | |  |
| I do not know my latest CD4 count at all | | | | | | | | | | | | | | | | | |  | |  | | | | | | | | | | | |  |
| I do not know my latest CD4 count, but the doctor said it was low | | | | | | | | | | | | | | | | | |  | |  | | | | | | | | | | | |  |
| I do not know my latest CD4 count, but the doctor said it was normal/fine | | | | | | | | | | | | | | | | | |  | |  | | | | | | | | | | | |  |
| Other | | | | | | | | | | | | | | | | | |  | |  | | | | | | | | | | | |  |
| **Q5 When was your latest CD4 test performed?** *COMPLETE WHAT IS APPLICABLE, E.G. 2 YRs 6 MNTHs* | | | | | | | | | | | | | | | | | | | | | | | | | | | | | | *DK* | | |
| Years | | | | |  | | |  | | | | | |  | | | | | | | | | | | | | | | |  | |  |
| Months | | | | |  | | |  | | | | | |  | | | | | | | | | | | | | | | |  | |  |
| Weeks | | | | |  | | |  | | | | | |  | | | | | | | | | | | | | | | |  | |  |
| Days | | | | |  | | |  | | | | | |  | | | | | | | | | | | | | | | |  | |  |
| **NCD History: The next few questions deal with chronic diseases like high blood pressure, diabetes and high cholesterol** | | | | | | | | | | | | | | | | | | | | | | | | | | | | | | | | |
| **Q6 Has a healthcare provider or doctor ever checked...** Your blood pressure? | | | | | | | | | | | | | | | | | | | | | | Y | | | | N | | | | DK | | |
| Your cholesterol or blood fats? | | | | | | | | | | | | | | | | | | | | | | Y | | | | N | | | | DK | | |
| Your blood sugar? | | | | | | | | | | | | | | | | | | | | | | Y | | | | N | | | | DK | | |
| **Q7 Have you ever been told by a doctor or other healthcare provider that you have HIGH BLOOD PRESSURE or HYPERTENSION?** | | | | | | | | | | | | | | | | | | | | | | Y | | | | N | | | | DK | | |
| **Q8 How long ago were you diagnosed with HIGH BLOOD PRESSURE or HYPERTENSION**  *COMPLETE WHAT IS APPLICABLE, E.G. 2 YRs 6 MNTHs* | | | | | | | | | | | | | | | | | | | | | | | | | | | | | | *DK* | | |
| Years | | | | |  | | |  | | | | | |  | | | | | | | | | | | | | | | |  | |  |
| Months | | | | |  | | |  | | | | | |  | | | | | | | | | | | | | | | |  | |  |
| Weeks | | | | |  | | |  | | | | | |  | | | | | | | | | | | | | | | |  | |  |
| Days | | | | |  | | |  | | | | | |  | | | | | | | | | | | | | | | |  | |  |
| **Q9 Where were you diagnosed with HIGH BLOOD PRESSURE or HYPERTENSION?** | | | | | | | | | | | | | | | | | | | | | | | | | | | | | | *DK* | | |
| Day hospital | | | | |  | | | | | | |  | | | | | | | | | | | | | | | | | |  | |  |
| Local clinic | | | | |  | | | | | | |  | | | | | | | | | | | | | | | | | |  | |  |
| Government hospital | | | | |  | | | | | | |  | | | | | | | | | | | | | | | | | |  | |  |
| TB clinic | | | | |  | | | | | | |  | | | | | | | | | | | | | | | | | |  | |  |
| ARV clinic | | | | |  | | | | | | |  | | | | | | | | | | | | | | | | | |  | |  |
| GP | | | | |  | | | | | | |  | | | | | | | | | | | | | | | | | |  | |  |
| Traditional healer | | | | |  | | | | | | |  | | | | | | | | | | | | | | | | | |  | |  |
| Pharmacy | | | | |  | | | | | | |  | | | | | | | | | | | | | | | | | |  | |  |
| Private hospital | | | | |  | | | | | | |  | | | | | | | | | | | | | | | | | |  | |  |
| Other | | | | |  | | | | | | |  | | | | | | | | | | | | | | | | | |  | |  |
| **Q10 Where do you most often receive healthcare or consult a healthcare professional for HIGH BLOOD PRESSURE or HYPERTENSION?** | | | | | | | | | | | | | | | | | | | | | | | | | | | | | | *DK* | | |
| Day hospital | | | | |  | | | | | | |  | | | | | | | | | | | | | | | | | |  | |  |
| Local clinic | | | | |  | | | | | | |  | | | | | | | | | | | | | | | | | |  | |  |
| Government hospital | | | | |  | | | | | | |  | | | | | | | | | | | | | | | | | |  | |  |
| TB clinic | | | | |  | | | | | | |  | | | | | | | | | | | | | | | | | |  | |  |
| ARV clinic | | | | |  | | | | | | |  | | | | | | | | | | | | | | | | | |  | |  |
| GP | | | | |  | | | | | | |  | | | | | | | | | | | | | | | | | |  | |  |
| Traditional healer | | | | |  | | | | | | |  | | | | | | | | | | | | | | | | | |  | |  |
| Pharmacy | | | | |  | | | | | | |  | | | | | | | | | | | | | | | | | |  | |  |
| Private hospital | | | | |  | | | | | | |  | | | | | | | | | | | | | | | | | |  | |  |
| Other | | | | |  | | | | | | |  | | | | | | | | | | | | | | | | | |  | |  |
| **Q11** ***Where else*** **do** **you** **receive** **healthcare or consult a healthcare professional for HYPERTENSION or HIGH BLOOD PRESSURE?** *TICK ALL THOSE APPLICABLE* | | | | | | | | | | | | | | | | | | | | | | | | | | | | | | *DK/NA* | | |
| Day hospital | | | | |  | | | | | | |  | | | | | | | | | | | | | | | | | |  | |  |
| Local clinic | | | | |  | | | | | | |  | | | | | | | | | | | | | | | | | |  | |  |
| Government hospital | | | | |  | | | | | | |  | | | | | | | | | | | | | | | | | |  | |  |
| TB clinic | | | | |  | | | | | | |  | | | | | | | | | | | | | | | | | |  | |  |
| ARV clinic | | | | |  | | | | | | |  | | | | | | | | | | | | | | | | | |  | |  |
| GP | | | | |  | | | | | | |  | | | | | | | | | | | | | | | | | |  | |  |
| Traditional healer | | | | |  | | | | | | |  | | | | | | | | | | | | | | | | | |  | |  |
| Pharmacy | | | | |  | | | | | | |  | | | | | | | | | | | | | | | | | |  | |  |
| Private hospital | | | | |  | | | | | | |  | | | | | | | | | | | | | | | | | |  | |  |
| Other | | | | |  | | | | | | |  | | | | | | | | | | | | | | | | | |  | |  |
| **Q12 Have you consulted a traditional healer for HIGH BLOOD PRESSURE or HYPERTENSION?** | | | | | | | | | | | | | | | | | | | | | | | | Y | | | | N | | | DK | |
| **Q13 Are you currently taking any herbal remedy or traditional medicine for HIGH BLOOD PRESSURE or HYPERTENSION?** | | | | | | | | | | | | | | | | | | | | | | | | Y | | | | N | | | DK | |
| **Q14 If a person has HIGH BLOOD PRESSURE or HYPERTENSION, what health consequences could arise if this is not treated?** *TICK ALL THOSE APPLICABLE* | | | | | | | | | | | | | | | | | | | | | | | | | | | | | | *DK* | | |
| None | |  | | | | | | | |  | | | | | | | | | | | | | | | | | | | | | |  |
| Death | |  | | | | | | | |  | | | | | | | | | | | | | | | | | | | | | |  |
| Heart attack | |  | | | | | | | |  | | | | | | | | | | | | | | | | | | | | | |  |
| Stroke | |  | | | | | | | |  | | | | | | | | | | | | | | | | | | | | | |  |
| Heart failure | |  | | | | | | | |  | | | | | | | | | | | | | | | | | | | | | |  |
| Amputations | |  | | | | | | | |  | | | | | | | | | | | | | | | | | | | | | |  |
| Kidney failure | |  | | | | | | | |  | | | | | | | | | | | | | | | | | | | | | |  |
| Cancer | |  | | | | | | | |  | | | | | | | | | | | | | | | | | | | | | |  |
| Infections | |  | | | | | | | |  | | | | | | | | | | | | | | | | | | | | | |  |
| HIV/AIDS | |  | | | | | | | |  | | | | | | | | | | | | | | | | | | | | | |  |
| Pregnancy complications | |  | | | | | | | |  | | | | | | | | | | | | | | | | | | | | | |  |
| Other (Specify) | |  | | | | | | | |  | | | | | | | | | | | | | | | | | | | | | |  |
| **Q15 What can a person do to prevent getting HIGH BLOOD PRESSURE or HYPERTENSION?** *TICK ALL THOSE APPLICABLE* | | | | | | | | | | | | | | | | | | | | | | | | | | | | | | *DK* | | |
| Lose Weight | |  | | | | | | | |  | | | | | | | | | | | | | | | | | | | | | |  |
| Quit smoking | |  | | | | | | | |  | | | | | | | | | | | | | | | | | | | | | |  |
| Do physical exercise | |  | | | | | | | |  | | | | | | | | | | | | | | | | | | | | | |  |
| Drink less alcohol | |  | | | | | | | |  | | | | | | | | | | | | | | | | | | | | | |  |
| Reduce fat in meals | |  | | | | | | | |  | | | | | | | | | | | | | | | | | | | | | |  |
| Increase salt in meals | |  | | | | | | | |  | | | | | | | | | | | | | | | | | | | | | |  |
| Eat less fresh fruit | |  | | | | | | | |  | | | | | | | | | | | | | | | | | | | | | |  |
| Eat more green leafy vegetables | |  | | | | | | | |  | | | | | | | | | | | | | | | | | | | | | |  |
| Other (Specify) | |  | | | | | | | |  | | | | | | | | | | | | | | | | | | | | | |  |
| **Q16 Have you ever been told by a healthcare provider that you have HIGH BLOOD SUGAR or DIABETES?** | | | | | | | | | | | | | | | | | | | | | | | | Y | | | | N | | | DK | |
| **Q17 How long ago were you diagnosed with HIGH BLOOD SUGAR or DIABETES?** | | | | | | | | | | | | | | | | | | | | | | | | | | | | | | *DK* | | |
| Years | | | |  | | |  | | | | | |  | | | | | | | | | | | | | | | | |  | |  |
| Months | | | |  | | |  | | | | | |  | | | | | | | | | | | | | | | | |  | |  |
| Weeks | | | |  | | |  | | | | | |  | | | | | | | | | | | | | | | | |  | |  |
| Days | | | |  | | |  | | | | | |  | | | | | | | | | | | | | | | | |  | |  |
| **Q18 Where were you diagnosed with HIGH BLOOD SUGAR or DIABETES?** | | | | | | | | | | | | | | | | | | | | | | | | | | | | | | *DK* | | |
| Day hospital |  | | | | | | | |  | | | | | | | | | | | | | | | | | | | | |  | |  |
| Local clinic |  | | | | | | | |  | | | | | | | | | | | | | | | | | | | | |  | |  |
| Government hospital |  | | | | | | | |  | | | | | | | | | | | | | | | | | | | | |  | |  |
| TB clinic |  | | | | | | | |  | | | | | | | | | | | | | | | | | | | | |  | |  |
| ARV clinic |  | | | | | | | |  | | | | | | | | | | | | | | | | | | | | |  | |  |
| GP |  | | | | | | | |  | | | | | | | | | | | | | | | | | | | | |  | |  |
| Traditional healer |  | | | | | | | |  | | | | | | | | | | | | | | | | | | | | |  | |  |
| Pharmacy |  | | | | | | | |  | | | | | | | | | | | | | | | | | | | | |  | |  |
| Private hospital |  | | | | | | | |  | | | | | | | | | | | | | | | | | | | | |  | |  |
| Other |  | | | | | | | |  | | | | | | | | | | | | | | | | | | | | |  | |  |
| **Q19 Where do you *most often* receive healthcare or consult a healthcare professional for HIGH BLOOD SUGAR or DIABETES?** | | | | | | | | | | | | | | | | | | | | | | | | | | | | | | *DK* | | |
| Day hospital |  | | | | | | | |  | | | | | | | | | | | | | | | | | | | | |  | |  |
| Local clinic |  | | | | | | | |  | | | | | | | | | | | | | | | | | | | | |  | |  |
| Government hospital |  | | | | | | | |  | | | | | | | | | | | | | | | | | | | | |  | |  |
| TB clinic |  | | | | | | | |  | | | | | | | | | | | | | | | | | | | | |  | |  |
| ARV clinic |  | | | | | | | |  | | | | | | | | | | | | | | | | | | | | |  | |  |
| GP |  | | | | | | | |  | | | | | | | | | | | | | | | | | | | | |  | |  |
| Traditional healer |  | | | | | | | |  | | | | | | | | | | | | | | | | | | | | |  | |  |
| Pharmacy |  | | | | | | | |  | | | | | | | | | | | | | | | | | | | | |  | |  |
| Private hospital |  | | | | | | | |  | | | | | | | | | | | | | | | | | | | | |  | |  |
| Other |  | | | | | | | |  | | | | | | | | | | | | | | | | | | | | |  | |  |
| **Q20** ***Where else*** **do** **you** **receive** **healthcare or consult a healthcare professional for HIGH BLOOD SUGAR or DIABETES** *TICK ALL THOSE APPLICABLE* | | | | | | | | | | | | | | | | | | | | | | | | | | | | | | *DK/NA* | | |
| Day hospital |  | | | | | | | |  | | | | | | | | | | | | | | | | | | | | |  | |  |
| Local clinic |  | | | | | | | |  | | | | | | | | | | | | | | | | | | | | |  | |  |
| Government hospital |  | | | | | | | |  | | | | | | | | | | | | | | | | | | | | |  | |  |
| TB clinic |  | | | | | | | |  | | | | | | | | | | | | | | | | | | | | |  | |  |
| ARV clinic |  | | | | | | | |  | | | | | | | | | | | | | | | | | | | | |  | |  |
| GP |  | | | | | | | |  | | | | | | | | | | | | | | | | | | | | |  | |  |
| Traditional healer |  | | | | | | | |  | | | | | | | | | | | | | | | | | | | | |  | |  |
| Pharmacy |  | | | | | | | |  | | | | | | | | | | | | | | | | | | | | |  | |  |
| Private hospital |  | | | | | | | |  | | | | | | | | | | | | | | | | | | | | |  | |  |
| Other |  | | | | | | | |  | | | | | | | | | | | | | | | | | | | | |  | |  |
| **Q21 Have you consulted a traditional healer for HIGH BLOOD SUGAR or DIABETES?** | | | | | | | | | | | | | | | | | | | | | | Y | | | | N | | | | DK | | |
| **Q22 Are you currently taking any herbal remedy or traditional medicine for HIGH BLOOD SUGAR or DIABETES?** | | | | | | | | | | | | | | | | | | | | | | Y | | | | N | | | | DK | | |
| **Q23 Have you ever been told by a doctor or other healthcare provider that you have HIGH CHOLESTEROL or HIGH BLOOD FATS?** | | | | | | | | | | | | | | | | | | | | | | Y | | | | N | | | | DK | | |
| **Q24 How long ago were you diagnosed with HIGH CHOLESTEROL or HIGH BLOOD FATS?** *COMPLETE WHAT IS APPLICABLE, E.G. 2 YRs 6 MNTHs* | | | | | | | | | | | | | | | | | | | | | | | | | | | | | | *DK* | | |
| Years | | |  | | |  | | | | | | |  | | | | | | | | | | | | | | | | |  | |  |
| Months | | |  | | |  | | | | | | |  | | | | | | | | | | | | | | | | |  | |  |
| Weeks | | |  | | |  | | | | | | |  | | | | | | | | | | | | | | | | |  | |  |
| Days | | |  | | |  | | | | | | |  | | | | | | | | | | | | | | | | |  | |  |
| **Q25 Where were you diagnosed with HIGH** **CHOLESTEROL or HIGH BLOOD FATS?** | | | | | | | | | | | | | | | | | | | | | | | | | | | | | | *DK* | | |
| Day hospital | | |  | | | | | | | |  | | | | | | | | | | | | | | | | | | |  | |  |
| Local clinic | | |  | | | | | | | |  | | | | | | | | | | | | | | | | | | |  | |  |
| Government hospital | | |  | | | | | | | |  | | | | | | | | | | | | | | | | | | |  | |  |
| TB clinic | | |  | | | | | | | |  | | | | | | | | | | | | | | | | | | |  | |  |
| ARV clinic | | |  | | | | | | | |  | | | | | | | | | | | | | | | | | | |  | |  |
| GP | | |  | | | | | | | |  | | | | | | | | | | | | | | | | | | |  | |  |
| Traditional healer | | |  | | | | | | | |  | | | | | | | | | | | | | | | | | | |  | |  |
| Pharmacy | | |  | | | | | | | |  | | | | | | | | | | | | | | | | | | |  | |  |
| Private hospital | | |  | | | | | | | |  | | | | | | | | | | | | | | | | | | |  | |  |
| Other | | |  | | | | | | | |  | | | | | | | | | | | | | | | | | | |  | |  |
| **Q26 Where do you *most often* receive healthcare or consult a healthcare professional for HIGH CHOLESTEROL or HIGH BLOOD FATS?** | | | | | | | | | | | | | | | | | | | | | | | | | | | | | | *DK* | | |
| Day hospital | | |  | | | | | | | |  | | | | | | | | | | | | | | | | | | |  | |  |
| Local clinic | | |  | | | | | | | |  | | | | | | | | | | | | | | | | | | |  | |  |
| Government hospital | | |  | | | | | | | |  | | | | | | | | | | | | | | | | | | |  | |  |
| TB clinic | | |  | | | | | | | |  | | | | | | | | | | | | | | | | | | |  | |  |
| ARV clinic | | |  | | | | | | | |  | | | | | | | | | | | | | | | | | | |  | |  |
| GP | | |  | | | | | | | |  | | | | | | | | | | | | | | | | | | |  | |  |
| Traditional healer | | |  | | | | | | | |  | | | | | | | | | | | | | | | | | | |  | |  |
| Pharmacy | | |  | | | | | | | |  | | | | | | | | | | | | | | | | | | |  | |  |
| Private hospital | | |  | | | | | | | |  | | | | | | | | | | | | | | | | | | |  | |  |
| Other | | |  | | | | | | | |  | | | | | | | | | | | | | | | | | | |  | |  |
| **Q27** ***Where else*** **do** **you** **receive** **healthcare or consult a healthcare professional for HIGH CHOLESTEROL or HIGH BLOOD FATS?** *TICK ALL THOSE APPLICABLE* | | | | | | | | | | | | | | | | | | | | | | | | | | | | | | *DK/NA* | | |
| Day hospital | | |  | | | | | | | |  | | | | | | | | | | | | | | | | | | |  | |  |
| Local clinic | | |  | | | | | | | |  | | | | | | | | | | | | | | | | | | |  | |  |
| Government hospital | | |  | | | | | | | |  | | | | | | | | | | | | | | | | | | |  | |  |
| TB clinic | | |  | | | | | | | |  | | | | | | | | | | | | | | | | | | |  | |  |
| ARV clinic | | |  | | | | | | | |  | | | | | | | | | | | | | | | | | | |  | |  |
| GP | | |  | | | | | | | |  | | | | | | | | | | | | | | | | | | |  | |  |
| Traditional healer | | |  | | | | | | | |  | | | | | | | | | | | | | | | | | | |  | |  |
| Pharmacy | | |  | | | | | | | |  | | | | | | | | | | | | | | | | | | |  | |  |
| Private hospital | | |  | | | | | | | |  | | | | | | | | | | | | | | | | | | |  | |  |
| Other | | |  | | | | | | | |  | | | | | | | | | | | | | | | | | | |  | |  |
| **Q28 Have you consulted a traditional healer for HIGH CHOLESTEROL/BLOOD FATS?** | | | | | | | | | | | | | | | | | | | | | | | | | Y | | | | N | | DK | |
| **Q29 Are you currently taking any herbal remedy or traditional medicine for HIGH CHOLESTEROL/BLOOD FATS?** | | | | | | | | | | | | | | | | | | | | | | | | | Y | | | | N | | DK | |
| **COMPLICATIONS OF NCDs**  **Q30 Have you ever had any of the following?** *PLEASE TICK THOSE CONDITIONS THAT APPLY* | | | | | | | | | | | | | | | | | | | | | | | | | | | | | | | | |
| Heart attack | | | | | | | | | | | | | | | | | | |  | |  | | | | | | | | |  | |  |
| Chest pain from heart disease or angina | | | | | | | | | | | | | | | | | | |  | |  | | | | | | | | |  | |  |
| Stroke, mild stroke or cerebrovascular accident | | | | | | | | | | | | | | | | | | |  | |  | | | | | | | | |  | |  |
| Kidney disease | | | | | | | | | | | | | | | | | | |  | |  | | | | | | | | |  | |  |
| Malignant tumour or cancer | | | | | | | | | | | | | | | | | | |  | |  | | | | | | | | |  | |  |
| Eye disease | | | | | | | | | | | | | | | | | | |  | |  | | | | | | | | |  | |  |
| Amputations | | | | | | | | | | | | | | | | | | |  | |  | | | | | | | | |  | |  |
| Vascular surgery or operations on the blood vessels to improve circulation | | | | | | | | | | | | | | | | | | |  | |  | | | | | | | | |  | |  |
| COPD or emphysema | | | | | | | | | | | | | | | | | | |  | |  | | | | | | | | |  | |  |
| **TUBERCULOSIS: The next few questions focus on TB  Q31 Have you ever been diagnosed with Tuberculosis (TB)?** | | | | | | | | | | | | | | | | | | | | | | | | | | | | | | *DK* | | |
| Yes, in childhood or many years ago | | | | | | | | | | | | | | | | | | |  | |  | | | | | | | | |  | |  |
| Yes, before I was diagnosed with HIV | | | | | | | | | | | | | | | | | | |  | |  | | | | | | | | |  | |  |
| Yes, after I was diagnosed with HIV | | | | | | | | | | | | | | | | | | |  | |  | | | | | | | | |  | |  |
| Yes, at the same time that I was diagnosed with HIV | | | | | | | | | | | | | | | | | | |  | |  | | | | | | | | |  | |  |
| No, but I am using prophylactic (preventative) medicine | | | | | | | | | | | | | | | | | | |  | |  | | | | | | | | |  | |  |
| No | | | | | | | | | | | | | | | | | | |  | |  | | | | | | | | |  | |  |
| **Q32 If you answered 'yes' to the previous question, have you been treated for TB since you were diagnosed with HIV?** | | | | | | | | | | | | | | | | | | | | | | | | | | | | | | | *DK* | |
| Yes, I was treated for TB after I was diagnosed HIV positive | | | | | | | | | | | | | | | |  |  | | | | | | | | | | | | |  | |  |
| No, I was treated for TB before I was diagnosed HIV positive | | | | | | | | | | | | | | | |  |  | | | | | | | | | | | | |  | |  |
| I cannot remember | | | | | | | | | | | | | | | |  |  | | | | | | | | | | | | |  | |  |
| **Q33 Where were you diagnosed with TB?** | | | | | | | | | | | | | | | | | | | | | | | | | | | | | | | | |
| Day hospital | | | | | | | | | | | | | |  |  | | | | | | | | | | | | | | |  | |  |
| Local clinic | | | | | | | | | | | | | |  |  | | | | | | | | | | | | | | |  | |  |
| Government hospital | | | | | | | | | | | | | |  |  | | | | | | | | | | | | | | |  | |  |
| TB clinic | | | | | | | | | | | | | |  |  | | | | | | | | | | | | | | |  | |  |
| ARV clinic | | | | | | | | | | | | | |  |  | | | | | | | | | | | | | | |  | |  |
| GP | | | | | | | | | | | | | |  |  | | | | | | | | | | | | | | |  | |  |
| Traditional healer | | | | | | | | | | | | | |  |  | | | | | | | | | | | | | | |  | |  |
| Pharmacy | | | | | | | | | | | | | |  |  | | | | | | | | | | | | | | |  | |  |
| Private hospital | | | | | | | | | | | | | |  |  | | | | | | | | | | | | | | |  | |  |
| Other | | | | | | | | | | | | | |  |  | | | | | | | | | | | | | | |  | |  |
| **Q34 Where do/did you most often receive healthcare or consult with a health professional for TB?** | | | | | | | | | | | | | | | | | | | | | | | | | | | | | | | | |
| Day hospital | | | | | | | | | | | | | |  |  | | | | | | | | | | | | | | |  | |  |
| Local clinic | | | | | | | | | | | | | |  |  | | | | | | | | | | | | | | |  | |  |
| Government hospital | | | | | | | | | | | | | |  |  | | | | | | | | | | | | | | |  | |  |
| TB clinic | | | | | | | | | | | | | |  |  | | | | | | | | | | | | | | |  | |  |
| ARV clinic | | | | | | | | | | | | | |  |  | | | | | | | | | | | | | | |  | |  |
| GP | | | | | | | | | | | | | |  |  | | | | | | | | | | | | | | |  | |  |
| Private hospital | | | | | | | | | | | | | |  |  | | | | | | | | | | | | | | |  | |  |
| Private pharmacy | | | | | | | | | | | | | |  |  | | | | | | | | | | | | | | |  | |  |
| Traditional healer | | | | | | | | | | | | | |  |  | | | | | | | | | | | | | | |  | |  |
| Other(specify) | | | | | | | | | | | | | |  |  | | | | | | | | | | | | | | |  | |  |
| **MEDICATION HISTORY: The next few questions focus on your medication** | | | | | | | | | | | | | | | | | | | | | | | | | | | | | | | | |
| **Q35 Can you list all the medication you take DAILY by name? Including any over-the-counter medicines, supplements, herbal remedies or traditional medicines** | | | | | | | | | | | | | | | | | | | | | | | Y | | | | N | | | **NA** | | |
| **Q36 Are you currently taking medication as prescribed by a doctor or other healthcare professional for the following medical conditions?** *PLEASE TICK THE CONDITIONS THAT APPLY* | | | | | | | | | | | | | | | | | | | | | | | | | | | | | | | | |
| High blood pressure or hypertension | | | | | | | | | | | | | |  |  | | | | | | | | | | | | | | |  | |  |
| High blood glucose or diabetes | | | | | | | | | | | | | |  |  | | | | | | | | | | | | | | |  | |  |
| High cholesterol or blood fats | | | | | | | | | | | | | |  |  | | | | | | | | | | | | | | |  | |  |
| HIV | | | | | | | | | | | | | |  |  | | | | | | | | | | | | | | |  | |  |
| TB | | | | | | | | | | | | | |  |  | | | | | | | | | | | | | | |  | |  |
| **Q37 Are you taking Aspirin/Disprin regularly to treat or prevent heart disease?** | | | | | | | | | | | | | | | | | | | | | | | Y | | | | N | | | DK | | |
| **Q38 Are you currently taking statins (anti-cholesterol medications like simvastatin, atorvastatin, lovastatin) regularly to treat or prevent heart disease?** | | | | | | | | | | | | | | | | | | | | | | | Y | | | | N | | | DK | | |
| **Q39 Do you collect/get all your medications from the same place?** | | | | | | | | | | | | | | | | | | | | | | | Y | | | | N | | | DK | | |
| **Q40 Where do you get/collect your medication for HIV?** | | | | | | | | | | | | | | | | | | | | | | | | | | | | | | *DK* | | |
| Day hospital | | | | | | | | | | | | | |  |  | | | | | | | | | | | | | | |  | |  |
| Local clinic | | | | | | | | | | | | | |  |  | | | | | | | | | | | | | | |  | |  |
| Government hospital | | | | | | | | | | | | | |  |  | | | | | | | | | | | | | | |  | |  |
| TB clinic | | | | | | | | | | | | | |  |  | | | | | | | | | | | | | | |  | |  |
| ARV clinic | | | | | | | | | | | | | |  |  | | | | | | | | | | | | | | |  | |  |
| GP | | | | | | | | | | | | | |  |  | | | | | | | | | | | | | | |  | |  |
| Private hospital | | | | | | | | | | | | | |  |  | | | | | | | | | | | | | | |  | |  |
| Private pharmacy | | | | | | | | | | | | | |  |  | | | | | | | | | | | | | | |  | |  |
| Traditional healer | | | | | | | | | | | | | |  |  | | | | | | | | | | | | | | |  | |  |
| At home, as part of a DOTS programme | | | | | | | | | | | | | |  |  | | | | | | | | | | | | | | |  | |  |
| Other(specify) | | | | | | | | | | | | | |  |  | | | | | | | | | | | | | | |  | |  |
| **Q41 Where do you get your medication for HIGH BLOOD PRESSURE or HYPERTENSION?** | | | | | | | | | | | | | | | | | | | | | | | | | | | | | | | *DK* | |
| Day hospital | | | | | | | | | | | | | |  |  | | | | | | | | | | | | | | |  | |  |
| Local clinic | | | | | | | | | | | | | |  |  | | | | | | | | | | | | | | |  | |  |
| Government hospital | | | | | | | | | | | | | |  |  | | | | | | | | | | | | | | |  | |  |
| TB clinic | | | | | | | | | | | | | |  |  | | | | | | | | | | | | | | |  | |  |
| ARV clinic | | | | | | | | | | | | | |  |  | | | | | | | | | | | | | | |  | |  |
| GP | | | | | | | | | | | | | |  |  | | | | | | | | | | | | | | |  | |  |
| Private hospital | | | | | | | | | | | | | |  |  | | | | | | | | | | | | | | |  | |  |
| Private pharmacy | | | | | | | | | | | | | |  |  | | | | | | | | | | | | | | |  | |  |
| Traditional healer | | | | | | | | | | | | | |  |  | | | | | | | | | | | | | | |  | |  |
| At home, as part of a DOTS programme | | | | | | | | | | | | | |  |  | | | | | | | | | | | | | | |  | |  |
| Other(specify) | | | | | | | | | | | | | |  |  | | | | | | | | | | | | | | |  | |  |
| **Q42 Where do you get your medication for HIGH BLOOD SUGAR or DIABETES?** | | | | | | | | | | | | | | | | | | | | | | | | | | | | | | *DK* | | |
| Day hospital | | | | | | | | | | | | | |  |  | | | | | | | | | | | | | | |  | |  |
| Local clinic | | | | | | | | | | | | | |  |  | | | | | | | | | | | | | | |  | |  |
| Government hospital | | | | | | | | | | | | | |  |  | | | | | | | | | | | | | | |  | |  |
| TB clinic | | | | | | | | | | | | | |  |  | | | | | | | | | | | | | | |  | |  |
| ARV clinic | | | | | | | | | | | | | |  |  | | | | | | | | | | | | | | |  | |  |
| GP | | | | | | | | | | | | | |  |  | | | | | | | | | | | | | | |  | |  |
| Private hospital | | | | | | | | | | | | | |  |  | | | | | | | | | | | | | | |  | |  |
| Private pharmacy | | | | | | | | | | | | | |  |  | | | | | | | | | | | | | | |  | |  |
| Traditional healer | | | | | | | | | | | | | |  |  | | | | | | | | | | | | | | |  | |  |
| At home, as part of a DOTS programme | | | | | | | | | | | | | |  |  | | | | | | | | | | | | | | |  | |  |
| Other(specify) | | | | | | | | | | | | | |  |  | | | | | | | | | | | | | | |  | |  |
| **Q43 Where do you get your medication for HIGH CHOLESTEROL or HIGH BLOOD FATS?** | | | | | | | | | | | | | | | | | | | | | | | | | | | | | | *DK* | | |
| Day hospital | | | | | | | | | | | | | |  |  | | | | | | | | | | | | | | |  | |  |
| Local clinic | | | | | | | | | | | | | |  |  | | | | | | | | | | | | | | |  | |  |
| Government hospital | | | | | | | | | | | | | |  |  | | | | | | | | | | | | | | |  | |  |
| TB clinic | | | | | | | | | | | | | |  |  | | | | | | | | | | | | | | |  | |  |
| ARV clinic | | | | | | | | | | | | | |  |  | | | | | | | | | | | | | | |  | |  |
| GP | | | | | | | | | | | | | |  |  | | | | | | | | | | | | | | |  | |  |
| Private hospital | | | | | | | | | | | | | |  |  | | | | | | | | | | | | | | |  | |  |
| Private pharmacy | | | | | | | | | | | | | |  |  | | | | | | | | | | | | | | |  | |  |
| Traditional healer | | | | | | | | | | | | | |  |  | | | | | | | | | | | | | | |  | |  |
| At home, as part of a DOTS programme | | | | | | | | | | | | | |  |  | | | | | | | | | | | | | | |  | |  |
| Other(specify) | | | | | | | | | | | | | |  |  | | | | | | | | | | | | | | |  | |  |
| **Q44 Where do/did you get your medication for TB?** | | | | | | | | | | | | | | | | | | | | | | | | | | | | | | *DK* | | |
| Day hospital | | | | | | | | | | | | | |  |  | | | | | | | | | | | | | | |  | |  |
| Local clinic | | | | | | | | | | | | | |  |  | | | | | | | | | | | | | | |  | |  |
| Government hospital | | | | | | | | | | | | | |  |  | | | | | | | | | | | | | | |  | |  |
| TB clinic | | | | | | | | | | | | | |  |  | | | | | | | | | | | | | | |  | |  |
| ARV clinic | | | | | | | | | | | | | |  |  | | | | | | | | | | | | | | |  | |  |
| GP | | | | | | | | | | | | | |  |  | | | | | | | | | | | | | | |  | |  |
| Private hospital | | | | | | | | | | | | | |  |  | | | | | | | | | | | | | | |  | |  |
| Private pharmacy | | | | | | | | | | | | | |  |  | | | | | | | | | | | | | | |  | |  |
| Traditional healer | | | | | | | | | | | | | |  |  | | | | | | | | | | | | | | |  | |  |
| At home, as part of a DOTS programme | | | | | | | | | | | | | |  |  | | | | | | | | | | | | | | |  | |  |
| Other(specify): | | | | | | | | | | | | | |  |  | | | | | | | | | | | | | | |  | |  |

| **MEDICATION ADHERENCE:** *Sometimes it is difficult to remember to take your medicine every day. Please answer the following questions about taking medication.* | | | | | | | | | | | | | |
| --- | --- | --- | --- | --- | --- | --- | --- | --- | --- | --- | --- | --- | --- |
| **Q45 How often do you...** | | | None of the time | Some of the time | | | Most of the time | All the time | DK | | N/A | | |
| Forget to take your HIV medication? | | |  |  | | |  |  |  | |  | | |
| Forget to take your medication for your other health problems? | | |  |  | | |  |  |  | |  | | |
| Decide not to take your HIV medication? | | |  |  | | |  |  |  | |  | | |
| Decide not to take your medication for your other health problems? | | |  |  | | |  |  |  | |  | | |
| Forget to attend scheduled health centre appointments? | | |  |  | | |  |  |  | |  | | |
| Decide not to attend scheduled health centre appointments? | | |  |  | | |  |  |  | |  | | |
| Leave the health centre without being attended to by a nurse or doctor? | | |  |  | | |  |  |  | |  | | |
| Leave the health centre without receiving your HIV medication? | | |  |  | | |  |  |  | |  | | |
| Leave the health centre without receiving medication for your other health problems? | | |  |  | | |  |  |  | |  | | |
| Stop taking your HIV medication because you don’t feel sick? | | |  |  | | |  |  |  | |  | | |
| Stop taking your medication for your other health problems because you don’t feel sick? | | |  |  | | |  |  |  | |  | | |
| Take someone else’s HIV medication? | | |  |  | | |  |  |  | |  | | |
| Take someone else’s medication for your other health problems? | | |  |  | | |  |  |  | |  | | |
| Not take your HIV medication because you don’t care or you think it’s not important? | | |  |  | | |  |  |  | |  | | |
| Not take your medication for your other health problems because you don’t care or you think it’s not important? | | |  |  | | |  |  |  | |  | | |
| **Q46 People often don't take medication exactly as it was prescribed by their doctor or health professional.**  **Do you ALWAYS take your HIV medication as prescribed?** | | | | | | | | | Y | N | | DK | |
| **Q47 If you answered 'NO' to the previous question, what was the main reason for not taking your HIV medication as it was prescribed?**  **………………………………………………………………………………………………………………………………..** | | | | | | | | | | | | | |
| **Q48 What were the possible other reasons for not taking your HIV medication as prescribed?** | | | | | | | | | | | | | |
| I felt better | | | | |  |  | | | | |  | |  |
| I was depressed | | | | |  |  | | | | |  | |  |
| I was not getting any better despite taking the medicines | | | | |  |  | | | | |  | |  |
| I felt worse after taking the medicines | | | | |  |  | | | | |  | |  |
| I was too busy with family obligations | | | | |  |  | | | | |  | |  |
| I didn’t like being different/separate from my family | | | | |  |  | | | | |  | |  |
| I had to look for a job for money | | | | |  |  | | | | |  | |  |
| I couldn’t afford transport to the clinic | | | | |  |  | | | | |  | |  |
| I had to go back to school | | | | |  |  | | | | |  | |  |
| My employer refused permission for me to go to the clinic | | | | |  |  | | | | |  | |  |
| I didn’t want to lose my job | | | | |  |  | | | | |  | |  |
| I couldn’t get enough time off work | | | | |  |  | | | | |  | |  |
| The clinic was too far | | | | |  |  | | | | |  | |  |
| The clinic ran out of medicine | | | | |  |  | | | | |  | |  |
| The doctors/nurses/clinic staff treated me badly | | | | |  |  | | | | |  | |  |
| I didn’t get enough support | | | | |  |  | | | | |  | |  |
| I was taking pills for too long | | | | |  |  | | | | |  | |  |
| I was taking too many pills | | | | |  |  | | | | |  | |  |
| The pills gave me side-effects | | | | |  |  | | | | |  | |  |
| A traditional healer told A friend/family member told me to stop me to stop | | | | |  |  | | | | |  | |  |
| I forgot to collect my medicines | | | | |  |  | | | | |  | |  |
| Other: | | | | |  |  | | | | |  | |  |
| **Q49 HIV TREATMENT CENTRE:  The next few questions relate to the health centre you attend for your HIV treatment. How would you rate the quality of healthcare you receive at your HIV treatment centre, specifically for HIV?** | | | | | | | | | | | | | |
| Very poor |  |  | | | | | | | | |  | |  |
| Poor |  |  | | | | | | | | |  | |  |
| Average |  |  | | | | | | | | |  | |  |
| Good |  |  | | | | | | | | |  | |  |
| Excellent |  |  | | | | | | | | |  | |  |

| **Q50 Why?**  **………………………………………………………………………………………………………………………………** | | | | | | | | | | | | | | | | | | |
| --- | --- | --- | --- | --- | --- | --- | --- | --- | --- | --- | --- | --- | --- | --- | --- | --- | --- | --- |
| **Q51 If you receive treatment for other health problems (like high blood pressure or diabetes) at the same health centre that you attend for HIV treatment, how would you rate the care you receive for your other health problems?** | | | | | | | | | | | | | | | | | | |
| Very poor |  |  | | | | | | | | | | | | |  | | |  |
| Poor |  |  | | | | | | | | | | | | |  | | |  |
| Average |  |  | | | | | | | | | | | | |  | | |  |
| Good |  |  | | | | | | | | | | | | |  | | |  |
| Excellent |  |  | | | | | | | | | | | | |  | | |  |
| Not Applicable |  |  | | | | | | | | | | | | |  | | |  |
| **Q52 Why?**  **………………………………………………………………………………………………………………………………** | | | | | | | | | | | | | | | | | | |
| **Q53 Are there any obstacles that make it difficult for you to receive your healthcare treatment?** *PLEASE SPECIFY YOUR MAIN OBSTACLE/REASON*  *:***………………………………………………………………………………………………………………………………** | | | | | | | | | | | | | | | | | | |
| **Q54 Are there any other obstacles that make it difficult for you to receive your healthcare?**  *PLEASE TICK ALL THOSE OPTIONS THAT APPLY.* | | | | | | | | | | | | | | | | | | |
| Healthcare visits are too expensive | | | | | |  |  | | | | | | | |  | | |  |
| I don’t have adequate transport | | | | | |  |  | | | | | | | |  | | |  |
| Transport is too expensive | | | | | |  |  | | | | | | | |  | | |  |
| I have previously been badly treated | | | | | |  |  | | | | | | | |  | | |  |
| I cannot take time off work | | | | | |  |  | | | | | | | |  | | |  |
| I have other commitments | | | | | |  |  | | | | | | | |  | | |  |
| The healthcare provider’s drugs or equipment are inadequate | | | | | |  |  | | | | | | | |  | | |  |
| The healthcare provider’s skills are inadequate | | | | | |  |  | | | | | | | |  | | |  |
| I have been denied healthcare by the facility staff | | | | | |  |  | | | | | | | |  | | |  |
| The healthcare provider told me I am not sick enough | | | | | |  |  | | | | | | | |  | | |  |
| Other (specify): | | | | | |  |  | | | | | | | |  | | |  |
| **Q55 How could the treatment of your HIV and other health problems (if you have any) be improved?** | | | | | | | | | | | | | | | | | | |
| Receiving treatment for all my health problems on the same day | | | | | | | |  |  | | | | | |  | | |  |
| Receiving treatment for all my health problems from the same healthcare provider | | | | | | | |  |  | | | | | |  | | |  |
| Receiving treatment for all my health problems at the same healthcare facility | | | | | | | |  |  | | | | | |  | | |  |
| More health facility staff | | | | | | | |  |  | | | | | |  | | |  |
| Shorter waiting times to see the healthcare provider (doctor/nurse) | | | | | | | |  |  | | | | | |  | | |  |
| Shorter waiting times at the pharmacy | | | | | | | |  |  | | | | | |  | | |  |
| Fewer tablets | | | | | | | |  |  | | | | | |  | | |  |
| Medication collection sites closer to where I live | | | | | | | |  |  | | | | | |  | | |  |
| Other (specify): | | | | | | | |  |  | | | | | |  | | |  |
| **FAMILY HISTORY**  **Q56 Do you have any blood relatives who suffer from the following conditions?** *PLEASE TICK THE OPTION(S) THAT APPLY.* | | | | | | | | | | | | | | | *DK* | | | |
| Diabetes or high blood sugar | | | | | |  |  | | | | | | | |  | | |  |
| High blood pressure or hypertension | | | | | |  |  | | | | | | | |  | | |  |
| High cholesterol or blood fats | | | | | |  |  | | | | | | | |  | | |  |
| Heart disease, heart attack or angina | | | | | |  |  | | | | | | | |  | | |  |
| Stroke | | | | | |  |  | | | | | | | |  | | |  |
| Cancer or malignant tumour | | | | | |  |  | | | | | | | |  | | |  |
| **PHYSICAL ACTIVITY:** The next questions are about the time you spend doing different types of physical activities. This includes activities you do at home, at work, travelling from place to place and during your spare time. Please answer the questions even if you don’t consider yourself to be an active person. | | | | | | | | | | | | | | | | | | |
| **Q57 Occupation-Related Physical Activity (paid or unpaid work):** When answering the following questions, think back over the **past 12 months** and think of **a usual week**.  Does your work involve vigorous activities, (like heavy lifting, digging, or heavy construction) for **at least 10 minutes** at a time? | | | | | | | | | | | | | Yes | | | No | | |
| **Q58** In **a usual week**, how many days do you do vigorous activities as part of your work?  Days: | | | | | | | | | | | | |  | | |  | | |
| **Q59** On **a usual day**, on which you do vigorous activities as part of your work, how much time do you spend doing such activities (hours and minutes)? | | | | | | | | | | | | | | | | | | |
| Hours: | | | | | | | | | | | | | |  | | |  | |
| Minutes: | | | | | | | | | | | | |  |  | | |  | |
| **Q60** Does your work involve moderate-intensity activities, (like brisk walking or carrying light loads) for **at least 10 minutes** at a time? | | | | | | | | | | | | | Yes | | | No | | |
| **Q61** In **a usual week**, how many days do you do moderate-intensity activities as part of your work? Days: | | | | | | | | | | | | |  | | |  | | |
| **Q62** On **a usual day**, on which you do moderate-intensity activities as part of your work, how much time do you spend doing such activities (hours and minutes)? | | | | | | | | | | | | | | | | | | |
| Hours: | | | | | | | | | | | | | |  | | |  | |
| Minutes: | | | | | | | | | | | | |  |  | | |  | |
| **Q63 Travel-Related Physical Activity:** The next questions ask you about the way you travel to and from places (to work, to shopping, to market, to church, etc.).  Do you walk or use a bicycle (pedal cycle) for **at least 10 minutes** at a time to get to and from places? | | | | | | | | | | | | | Yes | | | No | | |
| **Q64** In **a usual week**, on how many days do you walk or use a bicycle for at least 10 minutes at a time to get to and from places? Days: | | | | | | | | | | | | |  | | |  | | |
| **Q65** On **a usual day**, how much time do you spend walking or cycling for travel (hours and minutes)? | | | | | | | | | | | | | | | | | | |
| Hours: | | | | | | | | | | | | | |  | | |  | |
| Minutes: | | | | | | | | | | | | |  |  | | |  | |
| **Q66 Non-Work Related and Leisure Time Physical Activity:** The next questions ask about activities you do in your leisure or spare time, for recreation or fitness. Do not include the physical activities you do at work or for travel which have already been mentioned.  In your leisure or spare time, do you do any vigorous activities (like running or strenuous sports, weightlifting) for **at least 10 minutes** at a time? | | | | | | | | | | | | | Yes | | | No | | |
| **Q67** In **a usual week**, on how many days do you do vigorous activities for at least 10 minutes at a time during your leisure or spare time? Days: | | | | | | | | | | | | |  | | |  | | |
| **Q68** How much time do you spend doing vigorous activities as part of your leisure or spare time on a usual day when you do such activities a usual day? | | | | | | | | | | | | | | | | | | |
| Hours: | | | | | | | | | | | | | |  | | |  | |
| Minutes: | | | | | | | | | | | | |  |  | | |  | |
| **Q69** In your leisure or spare time, do you do any moderate-intensity activities (like brisk walking, cycling or swimming) for **at least 10 minutes** at a time? | | | | | | | | | | | | | Yes | | | No | | |
| **Q70** In **a usual week**, on how many days do you do moderate-intensity activities during your leisure or spare time? Days: | | | | | | | | | | | | |  | | |  | | |
| **Q71** On **a usual day**, when you do moderate-intensity physical activities during your leisure or spare time... How much time do you spend doing such activities? | | | | | | | | | | | | | | | | | | |
| Hours: | | | | | | | | | | | | | |  | | |  | |
| Minutes: | | | | | | | | | | | | |  |  | | |  | |
| **Q72 Sitting/Resting Activity:** The next questions ask you about the time spent sitting or resting, not including sleeping, **in the past 7 days**. This may include time sitting at a desk, visiting friends, reading, or sitting down to watch television **during working hours and leisure or spare time.**  Over the **past 7 days**, how much time did you spend sitting or reclining (lying) on **a usual day (excluding sleeping)**? | | | | | | | | | | | | | | | | | | |
| Hours: | | | | | | | | | | | | | |  | | |  | |
| Minutes: | | | | | | | | | | | | |  |  | | |  | |
|  | | | | | | | | | | | | | | | | | | |
| **SMOKING** | | | | | | | | | | | | | | | | | | |
| **Q73 Do you currently smoke any tobacco products such as cigarettes, cigars or pipes?** | | | | | | | | | | | | | | | Y | | | N |
| **Q74 Do you currently smoke any tobacco products daily?** | | | | | | | | | | | | | | | Y | | | N |
| **Q75 How old were you when you first started smoking daily?** Age in years | | | | | | | | | |  |  |  | | | DK | | | |
| **Q76 If you don't know how old you were when you started smoking daily, can you remember how long ago it was?** | | | | | | | | | | | | | | | *DK* | | | |
| Years | | |  |  |  | | | | | | | | | |  | | |  |
| Months | | |  |  |  | | | | | | | | | |  | | |  |
| Weeks | | |  |  |  | | | | | | | | | |  | | |  |
| Days | | |  |  |  | | | | | | | | | |  | | |  |

| **Q77 What do you smoke?** | | | | | | | | | | | | | | | | | | | | | | | |
| --- | --- | --- | --- | --- | --- | --- | --- | --- | --- | --- | --- | --- | --- | --- | --- | --- | --- | --- | --- | --- | --- | --- | --- |
| Commercial/manufactured cigarettes | |  | |  | | | | | | | | | | | | | | | | |  | |  |
| Hand-rolled cigarettes | |  | |  | | | | | | | | | | | | | | | | |  | |  |
| Pipes full of tobacco | |  | |  | | | | | | | | | | | | | | | | |  | |  |
| Cigars | |  | |  | | | | | | | | | | | | | | | | |  | |  |
| Cheroots | |  | |  | | | | | | | | | | | | | | | | |  | |  |
| Cigarillos | |  | |  | | | | | | | | | | | | | | | | |  | |  |
| Other (Specify): | |  | |  | | | | | | | | | | | | | | | | |  | |  |
| **Q78 On an average day, how much of these tobacco products do you smoke?** | | | | | | | | | | | | | | | | | | | | | | | |
| Manufactured cigarettes: | |  | |  | | |  | | | | | | | | | | | | | |  | |  |
| Hand-rolled cigarettes: | |  | |  | | |  | | | | | | | | | | | | | |  | |  |
| Pipes full of tobacco: | |  | |  | | |  | | | | | | | | | | | | | |  | |  |
| Cigars/Cheroots/Cigarillos: | |  | |  | | |  | | | | | | | | | | | | | |  | |  |
| Other: | |  | |  | | |  | | | | | | | | | | | | | |  | |  |
| **Q79 During the past 12 months, have you tried to stop smoking?** | | | | | | | | | | | | | | | | | | | | | Y | | N |
| **Q80 During a visit to a healthcare professional or doctor during the past 12 months, were** | | | | | | | | | | | | | | | | | | | | | | | |
| **you advised to stop smoking?** Yes | | | | | | | | | | | | | | | | | | | | |  | |  |
| No | | | | | | | | | | | | | | | | | | | | |  | |  |
| I cannot remember | | | | | | | | | | | | | | | | | | | | |  | |  |
| I have not visited a healthcare professional or doctor in the past 12 months | | | | | | | | | | | | | | | | | | | | |  | |  |
| **Q81 In the past, did you ever smoke tobacco products?** | | | | | | | | | | | | | | | | | | | | | Y | | N |
| **Q82 In the past, did you ever smoke tobacco products daily?** | | | | | | | | | | | | | | | | | | | | | Y | | N |
| **Q83 How old were you when you stopped smoking daily?** Age in years | | | | | | | | | | | | | | | |  |  |  | | | DK | | |
| **Q84 If you cannot recall how old you were when you stopped smoking daily, can you remember how long ago it was?** | | | | | | | | | | | | | | | | | | | | | *DK* | | |
| Years | |  |  | | |  | | | | | | | | | | | | | | |  | |  |
| Months | |  |  | | |  | | | | | | | | | | | | | | |  | |  |
| Weeks | |  |  | | |  | | | | | | | | | | | | | | |  | |  |
| Days | |  |  | | |  | | | | | | | | | | | | | | |  | |  |
| **Q85 Do you currently use smokeless tobacco in any form (snuff, chewing tobacco, betel)?** | | | | | | | | | | | | | | | | | | | | | Y | | N |
| **Q86 Do you currently use smokeless tobacco products daily?** | | | | | | | | | | | | | | | | | | | | | Y | | N |
| **Q87 If you answered 'yes' to the previous question... On average how many times a day do you use the following smokeless tobacco products?** | | | | | | | | | | | | | | | | | | | | | | | |
| Snuff by mouth | |  | |  | | | | | | | | | | | | | | | | |  | |  |
| Snuff by nose | |  | |  | | | | | | | | | | | | | | | | |  | |  |
| Chewing tobacco | |  | |  | | | | | | | | | | | | | | | | |  | |  |
| Betel/quid | |  | |  | | | | | | | | | | | | | | | | |  | |  |
| Other: | |  | |  | | | | | | | | | | | | | | | | |  | |  |
| **Q88 In the past, did you ever use any smokeless tobacco products (snuff, chewing tobacco, betel etc.)?** | | | | | | | | | | | | | | | | | | | | | Y | | N |
| **Q89 In the past, did you ever use smokeless tobacco products like snuff, chewing tobacco or betel daily?** | | | | | | | | | | | | | | | | | | | | | Y | | N |
| **Q90 How many people in your household smoke?** | | | | | | | | | | | | |  |  |  | | | | | | *DK* | | |
| **ALCOHOL INTAKE** | | | | | | | | | | | | | | | | | | | | | | | |
| **Q91 Have you consumed an alcoholic beverage in the past 12 months?** | | | | | | | | | | | | | | | | | | | Y | N | | DK | |
| **Q92 During the past 12 months, how often have you had at least one alcoholic drink?** | | | | | | | | | | | | | | | | | | | | | *DK* | | |
| Daily | |  | |  | | | | | | | | | | | | | | | | |  | |  |
| 5-6 days per week | |  | |  | | | | | | | | | | | | | | | | |  | |  |
| 1-4 days per week | |  | |  | | | | | | | | | | | | | | | | |  | |  |
| 1-3 days per month | |  | |  | | | | | | | | | | | | | | | | |  | |  |
| Less than once a month | |  | |  | | | | | | | | | | | | | | | | |  | |  |
| **Q93 During the past 30 days, on how many occasions did you have at least one alcoholic drink?** Number occasions: | | | | | | | | | | | | | | | | | | | | | *DK* | | |
| **Q94 During the past 30 days, when you drank alcohol, on average, how many standard alcoholic drinks did you have during one drinking occasion?**  (A standard drink is equivalent to 1 can (340ml) of beer, 1 glass (125ml) of wine, 1 shot (25ml) of spirits) | | | | | | | | | | | | | | | | | | | | | *DK* | | |
| **Q95 During the past 30 days, how many times did you have 6 or more standard alcoholic drinks in a single drinking occasion?** Number times: | | | | | | | | | | | | | | | | | | | | | *DK* | | |
| **Q96 During the past 7 days/1 week, how many standard alcoholic drinks did you have each day?** | | | | | | | | | | | | | | | | | | | | | | | |
| Monday |  |  | |  | | | | | | | | | | | | | | | | |  | |  |
| Tuesday |  |  | |  | | | | | | | | | | | | | | | | |  | |  |
| Wednesday |  |  | |  | | | | | | | | | | | | | | | | |  | |  |
| Thursday |  |  | |  | | | | | | | | | | | | | | | | |  | |  |
| Friday |  |  | |  | | | | | | | | | | | | | | | | |  | |  |
| Saturday |  |  | |  | | | | | | | | | | | | | | | | |  | |  |
| Sunday |  |  | |  | | | | | | | | | | | | | | | | |  | |  |
| **Q97 Is this amount of drinking typical of the past 12 months/1 year?** | | | | | | | | | | | | | | | | | | | | | *DK* | | |
| Yes, this is typical of the past 12 months | | | | |  | | |  | | | | | | | | | | | | |  | |  |
| No, this is less than the past 12 months | | | | |  | | |  | | | | | | | | | | | | |  | |  |
| No, this is more than the past 12 months | | | | |  | | |  | | | | | | | | | | | | |  | |  |
| **Q98 On average,** **how many standard drinks of the following did you consume on a typical day over the past week?** | | | | | | | | | | | | | | | | | | | | | | | |
| Homebrewed spirits (Moonshine/Mampoer) | | | | | | | | | | | | | | | | | | | | |  | |  |
| Homebrewed beer or wine | | | | | | | | | | | | | | | | | | | | |  | |  |
| Alcohol brought over the border, from another country | | | | | | | | | | | | | | | | | | | | |  | |  |
| Alcohol not intended for drinking (medicines containing alcohol, perfumes, after-shaves, methylated spirits) | | | | | | | | | | | | | | | | | | | | |  | |  |
| Untaxed alcohol | | | | | | | | | | | | | | | | | | | | |  | |  |
| **NUTRITION:** The following questions relate to your diet. Think of a typical day/week in the past year when answering these questions | | | | | | | | | | | | | | | | | | | | | | | |
| **Q99 In a typical week, on how many days do you eat fruit?** Days: | | | | | | | | | | | | | | | | | | | | |  | |  |
| **Q100 How many servings of fruit do you eat on one of those days?** | | | | | | | | | | | | | | | | | | | | | *DK* | | |
| 1-2 | | | | |  | | |  | | | | | | | | | | | | |  | |  |
| 3-4 | | | | |  | | |  | | | | | | | | | | | | |  | |  |
| 5-6 | | | | |  | | |  | | | | | | | | | | | | |  | |  |
| 7 or more | | | | |  | | |  | | | | | | | | | | | | |  | |  |
| **Q101 In a typical week, on how many days do you eat vegetables?** Days: | | | | | | | | | | | | | | | | | | | | |  | |  |
| **Q102 How many servings of vegetables do you eat on one of those days?** | | | | | | | | | | | | | | | | | | | | | *DK* | | |
| 1-2 | | | | |  | | |  | | | | | | | | | | | | |  | |  |
| 3-4 | | | | |  | | |  | | | | | | | | | | | | |  | |  |
| 5-6 | | | | |  | | |  | | | | | | | | | | | | |  | |  |
| 7 or more | | | | |  | | |  | | | | | | | | | | | | |  | |  |
| **Q103 How often do you add salt to your food before eating?** | | | | | | | | | | | | | | | | | | | | | | | |
| At every meal | | | | | | | | |  |  | | | | | | | | | | |  | |  |
| At occasional meals | | | | | | | | |  |  | | | | | | | | | | |  | |  |
| Most meals | | | | | | | | |  |  | | | | | | | | | | |  | |  |
| I taste the food to see whether I need to add salt | | | | | | | | |  |  | | | | | | | | | | |  | |  |
| I never add salt to my food before eating | | | | | | | | |  |  | | | | | | | | | | |  | |  |
| Other (Specify): | | | | | | | | |  |  | | | | | | | | | | |  | |  |
| **Q104 How salty do you like your food to be?** | | | | | | | | | | | | | | | | | | | | | *DK* | | |
| Not at all salty | | | | |  | | |  | | | | | | | | | | | | |  | |  |
| Lightly salted | | | | |  | | |  | | | | | | | | | | | | |  | |  |
| Very salty | | | | |  | | |  | | | | | | | | | | | | |  | |  |
| Other (Specify): | | | | |  | | |  | | | | | | | | | | | | |  | |  |
| **Q105 How do you think eating a lot of salt affects one's health?** | | | | | | | | | | | | | | | | | | | | | *DK* | | |
| Bad for health | | | | |  | | |  | | | | | | | | | | | | |  | |  |
| Good for health | | | | |  | | |  | | | | | | | | | | | | |  | |  |
| No effect on health | | | | |  | | |  | | | | | | | | | | | | |  | |  |
| Other (Specify): | | | | |  | | |  | | | | | | | | | | | | |  | |  |
| **Q106 Do you think that too much salt in your diet could cause a serious health problem?** | | | | | | | | | | | | | | | | | | | Y | N | | DK | |
| **Q107 How important is it to you to reduce the amount of salt in your diet?** | | | | | | | | | | | | | | | | | | | | | | | |
| Very important | | | | |  | | |  | | | | | | | | | | | | |  | |  |
| Important | | | | |  | | |  | | | | | | | | | | | | |  | |  |
| Neutral | | | | |  | | |  | | | | | | | | | | | | |  | |  |
| Not important | | | | |  | | |  | | | | | | | | | | | | |  | |  |
| I haven’t thought about it | | | | |  | | |  | | | | | | | | | | | | |  | |  |
| Other (Specify): | | | | |  | | |  | | | | | | | | | | | | |  | |  |
| **Q108 What type of milk do you usually use in your home?** | | | | | | | | | | | | | | | | | | | | | | | |
| Fresh whole (full-fat) | | | | |  | | |  | | | | | | | | | | | | |  | |  |
| Fresh 2% (low-fat) | | | | |  | | |  | | | | | | | | | | | | |  | |  |
| Fresh 1% (low-fat) | | | | |  | | |  | | | | | | | | | | | | |  | |  |
| Fresh skim (fat-free) | | | | |  | | |  | | | | | | | | | | | | |  | |  |
| UHT long-life full-fat | | | | |  | | |  | | | | | | | | | | | | |  | |  |
| UHT long-life 2% (low-fat) | | | | |  | | |  | | | | | | | | | | | | |  | |  |
| UHT long-life skim (fat-free) | | | | |  | | |  | | | | | | | | | | | | |  | |  |
| Powder, whole (full-fat) | | | | |  | | |  | | | | | | | | | | | | |  | |  |
| Powder, low-fat | | | | |  | | |  | | | | | | | | | | | | |  | |  |
| Powder fat-free (skim) | | | | |  | | |  | | | | | | | | | | | | |  | |  |
| Powder, blend | | | | |  | | |  | | | | | | | | | | | | |  | |  |
| Evaporated (tinned) | | | | |  | | |  | | | | | | | | | | | | |  | |  |
| Coffee creamer | | | | |  | | |  | | | | | | | | | | | | |  | |  |
| Other (Specify): | | | | |  | | |  | | | | | | | | | | | | |  | |  |
| **Q109 What type of butter or margarine is usually used in your home?** | | | | | | | | | | | | | | | | | | | | | *DK* | | |
| Brick/hard margarine | | | | |  | | |  | | | | | | | | | | | | |  | |  |
| Polyunsaturated margarine, tub | | | | |  | | |  | | | | | | | | | | | | |  | |  |
| Medium-fat spread/ "light" margarine | | | | |  | | |  | | | | | | | | | | | | |  | |  |
| Low-fat spread/ "extra-light" margarine | | | | |  | | |  | | | | | | | | | | | | |  | |  |
| Holsum | | | | |  | | |  | | | | | | | | | | | | |  | |  |
| Lard or suet | | | | |  | | |  | | | | | | | | | | | | |  | |  |
| Butter | | | | |  | | |  | | | | | | | | | | | | |  | |  |
| Ghee | | | | |  | | |  | | | | | | | | | | | | |  | |  |
| Other (Specify): | | | | |  | | |  | | | | | | | | | | | | |  | |  |
| **Q110 What type of oil is usually used in your home?** | | | | | | | | | | | | | | | | | | | | | *DK* | | |
| Sunflower oil | | | | |  | | |  | | | | | | | | | | | | |  | |  |
| Canola oil | | | | |  | | |  | | | | | | | | | | | | |  | |  |
| Olive oil | | | | |  | | |  | | | | | | | | | | | | |  | |  |
| Vegetable oil | | | | |  | | |  | | | | | | | | | | | | |  | |  |
| Peanut or other nut oil | | | | |  | | |  | | | | | | | | | | | | |  | |  |
| Avocado oil | | | | |  | | |  | | | | | | | | | | | | |  | |  |
| Grape seed oil | | | | |  | | |  | | | | | | | | | | | | |  | |  |
| Sesame oil | | | | |  | | |  | | | | | | | | | | | | |  | |  |
| Other (Specify): | | | | |  | | |  | | | | | | | | | | | | |  | |  |
| None | | | | |  | | |  | | | | | | | | | | | | |  | |  |
| **Q111 During the past 12 months has a doctor or other health professional advised you to do any of the following?** | | | | | | | | | | | | | | | | | | | | | | | |
| Quit using tobacco, or don’t start | | | | | | | | | | |  |  | | | | | | | | |  | |  |
| Reduce salt in your diet | | | | | | | | | | |  |  | | | | | | | | |  | |  |
| Eat at least 5 servings of fruit and/or vegetables per day | | | | | | | | | | |  |  | | | | | | | | |  | |  |
| Reduce fat in your diet | | | | | | | | | | |  |  | | | | | | | | |  | |  |
| Start to do more physical activity | | | | | | | | | | |  |  | | | | | | | | |  | |  |
| Maintain a healthy body weight or lose weight | | | | | | | | | | |  |  | | | | | | | | |  | |  |
| Other (Specify): | | | | | | | | | | |  |  | | | | | | | | |  | |  |
| None | | | | | | | | | | |  |  | | | | | | | | |  | |  |
| **Q112 Have you done any of the following over the past 12 months to stay healthy?** | | | | | | | | | | | | | | | | | | | | | | | |
| Lost weight if necessary | | | | | | | | | | |  |  | | | | | | | | |  | |  |
| Quit smoking | | | | | | | | | | |  |  | | | | | | | | |  | |  |
| Increased exercise | | | | | | | | | | |  |  | | | | | | | | |  | |  |
| Drank less alcohol | | | | | | | | | | |  |  | | | | | | | | |  | |  |
| Reduced fat in meals | | | | | | | | | | |  |  | | | | | | | | |  | |  |
| Reduced fried food | | | | | | | | | | |  |  | | | | | | | | |  | |  |
| Reduced salt in meals | | | | | | | | | | |  |  | | | | | | | | |  | |  |
| Ate more fresh fruit | | | | | | | | | | |  |  | | | | | | | | |  | |  |
| Ate more green leafy vegetables | | | | | | | | | | |  |  | | | | | | | | |  | |  |
| Other (please specify): | | | | | | | | | | |  |  | | | | | | | | |  | |  |

| **MEDICATION HISTORY** | | | | |
| --- | --- | --- | --- | --- |
| **Q113 List all the medication you take DAILY by name**  Include any over-the-counter medicines, supplements, herbal remedies or traditional medicines | | | | |
| LIST ALL MEDICATIONS TAKEN DAILY |  |  |  |  |
|  |  |  |  |  |
|  |  |  |  |  |
|  |  |  |  |  |
|  |  |  |  |  |
|  |  |  |  |  |
|  |  |  |  |  |
|  |  |  |  |  |
|  |  |  |  |  |
|  |  |  |  |  |
|  |  |  |  |  |
|  |  |  |  |  |
|  |  |  |  |  |
|  |  |  |  |  |
